# White-light crosslinkable milk protein bioadhesive with ultrafast gelation for first-aid wound treatment

**DOI:** 10.1186/s40824-023-00346-1

**Published:** 2023-02-03

**Authors:** Qinchao Zhu, Xuhao Zhou, Yanan Zhang, Di Ye, Kang Yu, Wangbei Cao, Liwen Zhang, Houwei Zheng, Ziyang Sun, Chengchen Guo, Xiaoqian Hong, Yang Zhu, Yajun Zhang, Ying Xiao, Teresa G. Valencak, Tanchen Ren, Daxi Ren

**Affiliations:** 1 Institute of Dairy Science, College of Animal Sciences, Zhejiang University, 310058 Hangzhou, China; 2 Department of Cardiology, Cardiovascular Key Laboratory of Zhejiang Province, Second Affiliated Hospital, School of Medicine, Zhejiang University, 310027 Hangzhou, China; 3 Key Laboratory of Animal Virology of Ministry of Agriculture, Center for Veterinary Sciences, Zhejiang University, 310058 Hangzhou, China; 4 Department of Veterinary Medicine, College of Animal Sciences, Zhejiang University, 310058 Hangzhou, China; 5 Key Laboratory of 3D Printing Process and Equipment of Zhejiang Province, School of Mechanical Engineering, Zhejiang University, 310027 Hangzhou, China; 6 MOE Key Laboratory of Macromolecular Synthesis and Functionalization, Department of Polymer Science and Engineering, Zhejiang University, 310027 Hangzhou, China; 7 School of Engineering, Westlake University, 310023 Hangzhou, Zhejiang China; 8 Sir Run Run Shaw Hospital, School of Medicine, Zhejiang University, 310020 Hangzhou, Zhejiang China

**Keywords:** Casein, photocrosslink, Bioadhesive, Hemostasis, Wound healing

## Abstract

**Background:**

Post-traumatic massive hemorrhage demands immediately available first-aid supplies with reduced operation time and good surgical compliance. *In-situ* crosslinking gels that are flexibly adapting to the wound shape have a promising potential, but it is still hard to achieve fast gelation, on-demand adhesion, and wide feasibility at the same time.

**Methods:**

A white-light crosslinkable natural milk-derived casein hydrogel bioadhesive is presented for the first time. Benefiting from abundant tyrosine residues, casein hydrogel bioadhesive was synthesized by forming di-tyrosine bonds under white light with a ruthenium-based catalyst. We firstly optimized the concentration of proteins and initiators to achieve faster gelation and higher mechanical strength. Then, we examined the degradation, cytotoxicity, tissue adhesion, hemostasis, and wound healing ability of the casein hydrogels to study their potential to be used as bioadhesives.

**Result:**

Rapid gelation of casein hydrogel is initiated with an outdoor flashlight, a cellphone flashlight, or an endoscopy lamp, which facilitates its usage during first-aid and minimally invasive operations. The rapid gelation enables 3D printing of the casein hydrogel and excellent hemostasis even during liver hemorrhage due to section injury. The covalent binding between casein and tissue enables robust adhesion which can withstand more than 180 mmHg blood pressure. Moreover, the casein-based hydrogel can facilitate post-traumatic wound healing caused by trauma due to its biocompatibility.

**Conclusion:**

Casein-based bioadhesives developed in this study pave a way for broad and practical application in emergency wound management.

**Supplementary Information:**

The online version contains supplementary material available at 10.1186/s40824-023-00346-1.

## Introduction

Uncontrollable massive hemorrhage following trauma or occurring during surgery accounts for a significant proportion of annual mortality worldwide [1]. The traditional mechanical products and technologies for wound closure, viz. sutures, tapes, staples and clips, are used to speed up wound healing based on physical juxtaposition of wound edges [2, 3]. However, they require a mass of user training and proper equipment and the process is also time-consuming [4]. Developing fast and effective wound hemostatic bioadhesive may become very useful to induce post-traumatic hemostasis in clinical and surgical operations [2, 5]. To achieve desirable therapeutic effects, an ideal bioadhesive should have proper mechanical properties, biocompatibility, non-toxicity, strong adhesion capacity on target surface, and short cross-linking duration [6, 7].

A number of different bioadhesives have been investigated for skin wound closure to replace sutures or wound dressings, such as fibrin gel, cyanoacrylates and polyethylene glycol (PEG)-based adhesives [8, 9]. However, the functional properties of these adhesives are somewhat suboptimal, for example, they may cause excessive inflammation and necrosis, have a slow curing rate, a weak adhesive strength or/and a high swelling ratio [10–12]. Many new bioadhesives have been developed recently, and reportedly have sound biocompatibility, reliable bio-adhesion, sufficient mechanical properties and the ability to suppress bacterial growth while promoting healing by combining multiple functional compositions together. Xu et al. designed hyaluronic acid- and catechol-based adhesive hydrogels with fast gelation to adhere to the stomach [13]. Lei et al. developed an injectable and recoverable dual-network (DN) hydrogels with inherently antibacterial properties and composed of poly(l-lysine)-graft-4-hydroxyphenylacetic acid (PLL-g-HPA) and Aga, an enzyme-adjusted cross-linkage reaction and a strong H-bonding [14]. Zou et al. developed a multifunctional hydrogel comprising of polysaccharides and tannic acid through a multi-crosslinking strategy to effectively promoted the healing of infected wounds [15]. However, the on-site mixture of multiple components may increase variation in material properties during emergency situation.

Photocrosslinkable bioadhesives have raised broad interest due to their advantages in on-demand controllable crosslinking reactions for *in-situ* adaptive wound closure. Despite the fact that UV-curable systems were applied traditionally, systems using visible light become more attractive due to the hazard involved with UV [16]. Moreover, the light sources of longer wave length offer a significantly deeper light penetration compared to the UV light or blue light, and allowing adhesion for deep wounds [17]. The white-light photocrosslinkable approach bears smaller risk in secondary damage to either tissues and eyes and all caused by free radicals and oxidative stress from UV overexposure [18]. White-light crosslinking is convenient enough as not require for device conversion. The light source can be outdoor flashlight, the cellphone flashlight for first-aid treatment, or the lamp attached to endoscopy for minimal invasive therapy.

Ruthenium/sodium persulfate has recently been developed as a promising white-light photoinitiator [19]. In the presence of white light and SPS, Ru mainly acts on the tyrosine group, which is further converted to free radical of tyrosine forming covalent di-tyrosine bonds with the nearby tyrosine radical [20]. At present, Ru-based photoinitiators have been used to cross-link Collagen-, fibrin-and silk fibroin-based natural biomaterials and shown great biocompatibility [21–23]. Besides, the high molar extinction coefficient of Ru, enables a good curing effect at relatively low initiator concentration, further reduced the potential toxicity [24]. The tyrosine present on the tissue surface can also react with the adhesive to form di-tyrosine bonds and augment the interfacial bonding strength [25]. We speculate Ru/SPS system can be applied as photoinitiator to create a white-light controllable bioadhesive by combining with a suitable polymer.

Casein is the mainly protein (80% of total protein) in cow’s milk, it contains abundant tyrosine groups, which may promote the rapid cross-linking of casein to form stable bioadhesive under Ru/SPS system. Nearly 0.25 billion tons of casein was produced by cows per year, most of them were consumed in dairy products such as milk and cheese, or as ingredient used in food industry. Except that casein was also popular in papermaking, leather, construction, plastics and forming industry due to their good performance of adhesiveness, brightness, stability, emulsibility [26, 27], and low price (about $5/kg). On the other hand, casein has many desirable properties as potential biomaterial, including biocompatibility, biodegradability and low immunogenicity [28], may attract great attention in material area in future.

**Fig. 1:**
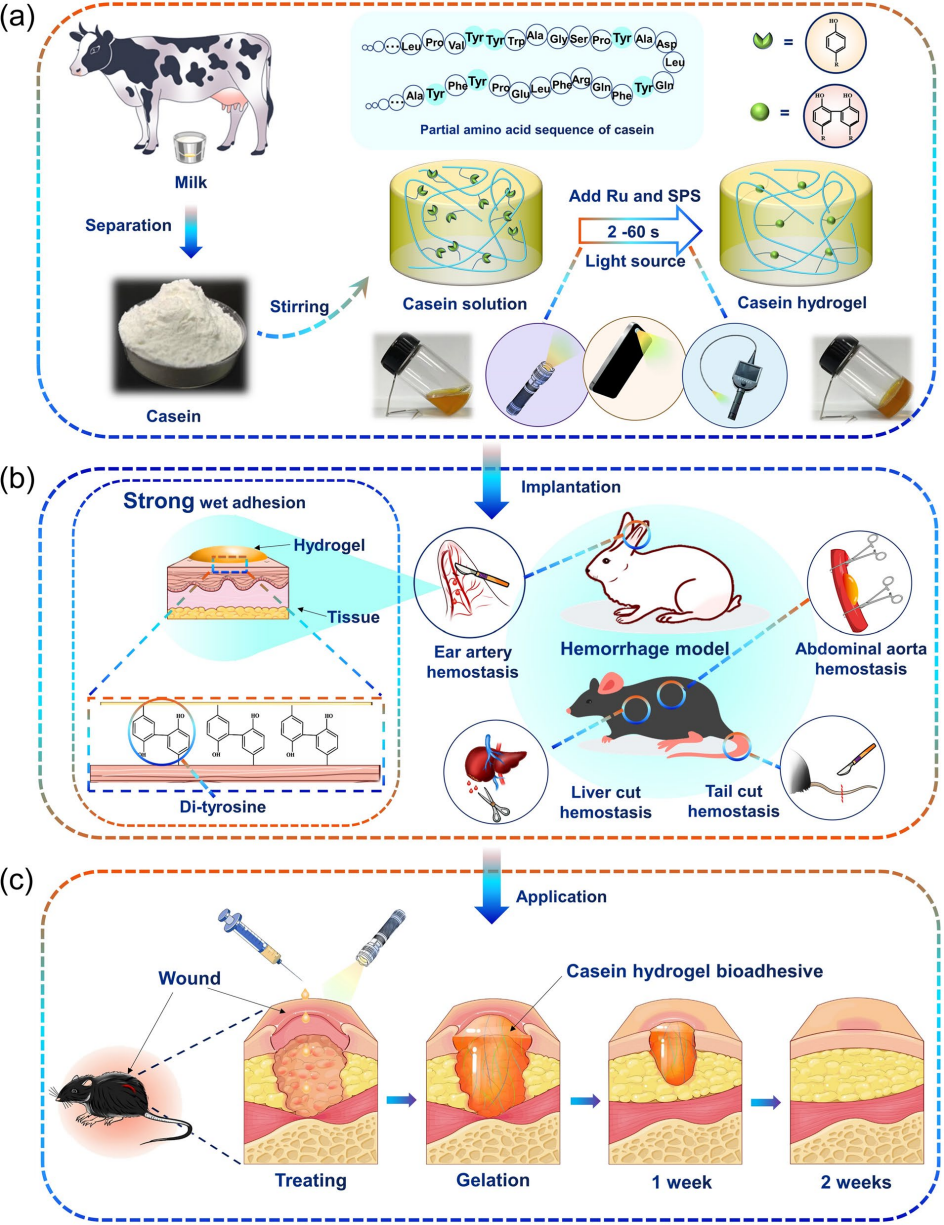
Schematic illustration of the preparation and the reaction process of casein hydrogels which accelerates hemostasis and wound healing. (**a**) Casein from milk can be crosslinked through white-light activated di-tyrosine bonds formation initiated by Ru/SPS to form hydrogel; (**b**) Adhesion mechanism and hemostatic property of the casein hydrogel bioadhesive for massive arterial and visceral hemorrhage; (**c**) The process of wound healing with casein hydrogel bioadhesive treatment

In this work, we integrated Ru/SPS redox system with casein for the first time to develop a milk derived bioadhesive as a first-aid tissue adhesive with ultrafast wound hemostasis. Upon visible light exposure, the casein-based hydrogel was crosslinked by the reaction between tyrosine residues (Fig. 1a). Covalent tissue adhesive was achieved due to the formation of the di-tyrosine bond at casein–tissue interface (Fig. 1b). Wound closure property and hemostatic property of the casein-based bioadhesive was evaluated by various large dose hemorrhage models (Fig. 1b). In vitro and in vivo wound healing experiments were carried to study the biocompatibility, biodegradability and wound healing promoting ability of the casein-based bioadhesive (Fig. 1c). This study shed new light on the development of bioadhesive based on new sources and simple strategies. This single component casein-based bioadhesive could emerge as a promising first-aid supply for trauma management.

## Experimental section

### Synthesis of casein hydrogel

For white light cross-linking process, the sodium persulfate (SPS) (Sinopharm Chemical Reagent Co., Ltd., Shanghai, China), tris(2,2-bipyridyl) dichlororuthenium (II) hexahydrate (Ru) (Xianding, Shanghai, China) and casein (Fonterra, Shanghai, China) were dissolved in 0.1 M NaOH solution under stirring, and the pH was then adjusted to 7.8. The samples were irradiated with a white LED lamp (15 W) from a distance of 1 cm for 3 min to ensure complete gelation.

### Morphological characterization

The casein hydrogels (5% w/v, 10% w/v, 15% w/v) (8 mm diameter and 6 mm height) were prepared, washed three times with MilliQ water, and freeze-dried under − 20 °C. Cross-sections of lyophilized hydrogels were exposed, sprayed with Au film to provide a conductive environment, and observed by an inverted optical microscopy and a field-emission scanning electron microscopy (SEM) (GeminiSEM 300, Carl Zeiss, Germany). Image J software was employed to measure the pore sizes of hydrogel samples. For each hydrogel sample (at least 3 samples), every hydrogel had three pictures taken from various regions of the hydrogel. Every picture was measured by more than ten pores. The above data were processed using Origin 2018 for normal distribution analysis.

### FTIR spectra

Freeze-dried casein hydrogel and raw casein powder were scanned by a FTIR spectrophotometer (Thermo Scientific Nicolet iS20, Thermo Electron Co., Waltham, MA, USA), at 32 times per second, from 4000 to 400 cm^− 1^.

### Rheology of casein hydrogel

The dynamic storage (G′) and loss modulus (G″) during casein gelation were measured by a rheometer (MCR302, Anton Paar, Austria) equipped with a Peltier element for temperature control and a generator. The casein solutions (5% w/v, 10% w/v, 15% w/v) with various Ru (0.2, 0.5, 1, 2 mM) and SPS (20, 30, 40, 60, 80 mM) concentration were placed between the plates at 37 °C to completely fill the gap (0.5 mm). Under 1.5 mW cm^− 2^, 450 nm visible irradiation, time sweep oscillatory measurements were performed at a frequency of 30 Hz and 1% strain. The point where G′ and G′′ intersect was considered to be the gelation point; the point where the elastic modulus reaches a plateau is considered as complete crosslinking.

In addition, to further explore the effect of light intensity on the gelation rate of casein hydrogels, the mixed solutions (10%/1 mM/40 mM casein/Ru/SPS) were placed between the plates at 37 °C to completely fill the gap (0.5 mm) and gel under 450 nm visible irradiation of different light intensities from 1.5 mW cm^− 2^ to 40.2 mW cm^− 2^.

### Mechanical test

The hydrogel with an 8 mm diameter and 6 mm height was crosslinked with a white LED lamp (15 W) from a distance of 1 cm for 3 min. The strain-stress curve was obtained via a mechanical tester (Instron 5543 A, America) with a compression rate at 1 mm/min, which has a load cell with a maximum range of 500 N. The linear region of the compressive curve was defined as the elastic region, and the slope of the linear region (when the strain was below 5%) was calculated as the compressive modulus. Three parallel samples were measured to calculate the average values.

### Swelling test

The initial weight of casein hydrogels (5% w/v, 10% w/v, 15% w/v casein) (8 mm diameter and 6 mm height) was accurately measured after being fabricated. Then, the hydrogel samples were immersed in 15 mL PBS buffer (pH = 7.4) at 25 °C and were swollen until the equilibrium state was reached. After specified time intervals, the swollen hydrogels were weighted after gently removing excess water using filter paper. The swelling ratio was defined as follows:

$$\text{s}\text{w}\text{e}\text{l}\text{l}\text{i}\text{n}\text{g} \text{r}\text{a}\text{t}\text{i}\text{o}=\frac{{m}_{i}-{m}_{0}}{{m}_{0}}$$

Where m_i_ and m_0_ were represent the weights of the swollen and initial samples, respectively.

After soaking in PBS for 168 h, the casein hydrogels (5% w/v, 10% w/v, 15% w/v) were lyophilized under − 20 °C. Cross-sections of lyophilized hydrogels were exposed, sputter-coated with gold, and observed by an inverted optical microscopy and a field-emission SEM (GeminiSEM 300, Carl Zeiss, Germany).

### Digital light processing 3D printing

3D printing has been carried out as in our previous studies [29]. DLP photopatterning experiments were conducted using a high-quality DLP printer for laboratory use. Our DLP printing system consisted of three major components: a UV Digital Micro-mirror Device™ with 30 μm resolution (1920 × 1068 × 1080 pixels; Texas Instruments, Dallas, USA), a 405 nm UV-LED (LG Innotec, Seoul Korea) with on average 12 mW cm^− 2^ intensity, and a lens module (focal length 60 mm, aperture f/2.8, distortion < 80%, offset 0%, working distance 130 mm, and field of view 28.8 × 16.5 mm) with two UV-grade biconvex lenses (24 mm diameter). The build area was 35 (L) ×20 (W) × 120 (H) mm with a layer thickness adjustable from 5 to 200 μm. The system was customized by professional manufacturers (NBRTech. Ltd, Chuncheon, Korea and Illuminaid. Ltd, Seongnam, Korea). The 3D molds with desired structures were designed, converted and sliced as described in our previous study [30]. An amount of 10% w/v casein precursor solution with 1/40 mM Ru/SPS was placed onto the print station. The printing parameters were set as: printing thickness 50 μm, exposure time 30 s, light intensity 12 mW cm^− 2^. After printing, the constructs were rinsed with PBS to remove the un-crosslinked solution. Printed constructs were further photographed under a microscope.

### Adhesion of casein hydrogel bioadhesive on wet tissues

Two 5 mm-diameter circular wounds were created on the damaged porcine heart, from which water could flow out. Precursor solution of 10% casein hydrogel bioadhesive was added to a wound and gelled *in-situ* for 50 s using an ordinary bright flashlight, and the wound sealing was observed under water flow. Casein hydrogel bioadhesives gelled *in-situ* on porcine skin and liver for 20 s using an ordinary bright flashlight and were observed under distorting, bending, or water flushing. Moreover, tow porcine skins were adhered with casein hydrogel bioadhesive, which was immersed in PBS for 20 h.

In addition, 100 µL of the casein and photoinitiator mixture was applied onto a piece of 4 × 6 cm^2^ porcine skin, after which the hydrogels formed in situ after visible light illumination using cellphone flight for 30 s and endoscopy lamp for 60 s, respectively. The casein hydrogel bioadhesives were observed under distorting.

To measure the shear strength and tensile strength of photocrosslinked casein hydrogel bioadhesives and fibrin gel, two pieces of porcine skin (2 cm width and 4 cm length) with an overlap area of width 2 cm and length 1 cm or edge to edge were adhered through hydrogel bioadhesives formed *in-situ* for 20 s using an ordinary bright flashlight, which was subjected to a tensile test using a mechanical tester (Instron 5543 A). All tests were performed at a constant stretching speed of 10 mm min^− 1^. Shear strength was determined by dividing the maximum force by the adhesion area. Tensile strength was determined by dividing the maximum force by the bond area.

**Fig. 2:**
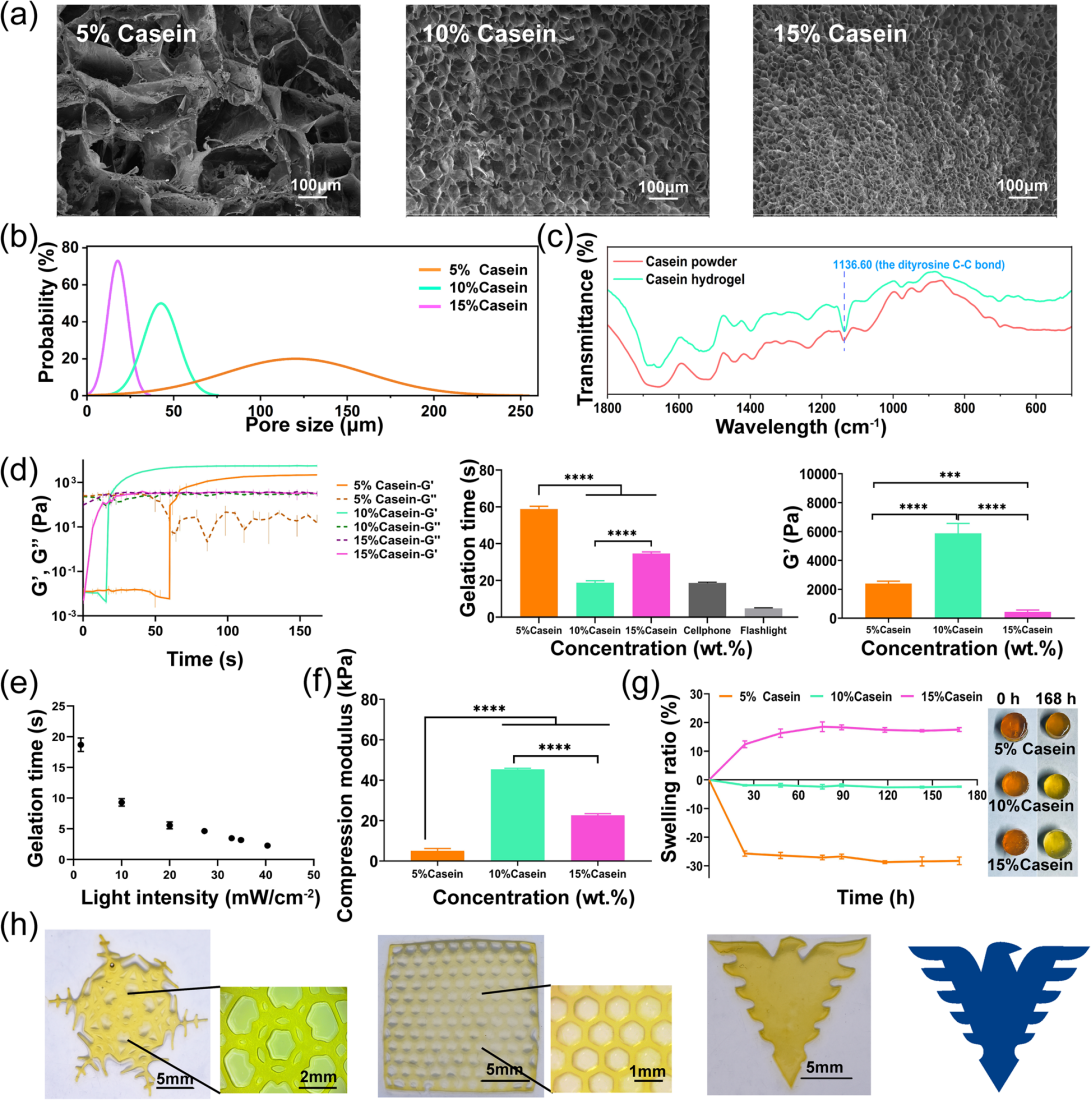
Casein hydrogels characterization. (**a**) SEM images of different hydrogels, scale bar: 100 μm; (**b**) Pore size distribution of different hydrogels; (**c**) FTIR spectrum of casein powder and casein hydrogels; (**d**) Rheology analyses, gelation time and storage modulus of casein solution with different concentrations (5%, 10%, 15%), upon exposure to visible light at 450 nm (*n* = 3); (**e**) Gelation time of casein hydrogels with different light intensity at 450 nm; (**f**) Compression modulus of casein solution concentrations (5%, 10%, 15%) (*n* = 3), (**g**) Mass swelling ratio with casein hydrogels (5%, 10%, 15%) at different time point (mean ± SD; *n* = 3); (**h**) Snowflake, mesh and eagle patterned hydrogels prepared by 3D printing

To measure the burst pressure of photocrosslinked casein hydrogel bioadhesives and fibrin gel, a piece of porcine skin was held in a chamber attached to a syringe. The bioadhesive was formed *in-situ* for 10 s using an ordinary bright flashlight on the surface of porcine skin, covering a 2-mm diameter hole, after which the syringe started pumping the PBS (pH 7.4, 37 °C) solution and applying pressure to the bioadhesive-sealed hole (Fig. 2c). the pressure on the bioadhesives were measured with a digital manometer. The value at which the pressure began to decrease was considered to be the peak pressure. All experiments were repeated three times.

### Cytotoxicity of casein hydrogel bioadhesive

Bioadhesive cytotoxicity was evaluated with a previously described method [31]. In brief, the hydrogel with an 8 mm diameter and 6 mm height was crosslinked with a white LED lamp (15 W) from a distance of 1 cm for 3 min. The extracts were prepared by immersed bulk casein hydrogel bioadhesive with various SPS concentrations (30 mM, 40 mM, 60 Mm, 80 mM) in a DMEM medium containing 10% FBS at an extraction ratio of 200 mg/ml for 12 h at 37 °C. The L929 mice fibroblast (5 × 10^3^ cells per well) were seeded in 96-well cell culture plates. Then, the culture media was replaced by 100 µL extract, while fresh medium was substituted in the blank group. After 24 h, cell viability was tested by Cell Counting Kit-8 (Yeasen Biotech, China) and Live/Dead staining kit (Yeasen Biotech, China) following manufacturer’s instructions. For CCK8 assay, the absorbance at 450 nm of the media was measured after 2 h by a microplate reader (Tecan M200 PRO, Tecan Company, Switzerland). For Live/Dead assay, images were captured by a fluorescence microscope (Leica DMI8, Germany), and further analyzed by ImageJ software to count the ratio of live cells.

### In vitro coagulation test

For each biological repetition, blood for every group was collected from male C57BL/6 N mice aged 10 weeks and weighing 25 ± 2.5 g. After the mice were anesthetized with pentobarbital, 50 µL of fresh blood were immediately taken by tail clipping and mixed with 100 µL of the material in each test tube. After irradiated with an outdoor flashlight for 1 min to gel, 1 ml ddH_2_O was added along the tube wall to mix gently without blowing off the blood clot to make the color of the solution uniform. 100 µL supernatant were pipetted from each tube into a 96-well plate, and the absorbance at 540 nm of the supernatant was measured by a microplate reader. The positive reference was the mixture of fresh blood and ddH_2_O, and the endogenous coagulation reference was the mixture of blood clots and ddH_2_O after natural coagulation. All tests were repeated 5 times for each group.

The blood clotting index (BCI) is defined as follows:

$$\text{B}\text{C}\text{I}=\frac{{OD}_{i}}{{OD}_{0}}\times 100\%$$

where OD_i_ represent the OD_540_ of the natural coagulation group, gauze-treated group, casein-treated group and fibrin gel, and OD_0_ represent the OD_540_ of positive reference.

### Hemostasis performance of bioadhesives

All animal operations and experimental procedures were approved by the Ethics Committee of Zhejiang University (Application Number:17,426). Male C57BL/6 N mice, aged 8 weeks and weighing 20 ± 2.5 g were purchased from Zhejiang Vital River Laboratory Animal Technology Co., Ltd for following mouse experiments. First, an anesthetized mouse was fixed in position and 1/3 of the tail was clipped with surgical scissors. The wound was exposed in air for 10 s to ensure normal bleeding. Then, the hydrogel precursor solution was immediately added dropwise to the tail with light irradiation for 10 s to crosslink *in-situ*, and the blood was absorbed with filter paper. After 10 min, the weight of the sample and filter paper were measured. The blank group received no treatment after tail clipping. All tests were repeated 5 times for each group.

For mouse liver hemorrhage experiments, a pre-weighed filter paper was placed beneath the left lobe of the liver, and bleeding was induced by a 15 mm incision. Casein hydrogel precursor solution was dropped on liver wounds and gelled using an ordinary bright flashlight for 45 s. The hemostatic effects of the casein hydrogel bioadhesive and fibrin gel-treated groups were recorded, while the untreated group served as a blank. After 2 min, the weight of the sample and filter paper were measured. All experiments were repeated 5 times and recorded by camera for each group.

For ear artery hemorrhage experiments, 6 male New Zealand Rabbits weighing about 2.5 kg were used in this experiment. Animals were sedated with propofol and maintained on 2–3% isoflurane. Rabbits were randomly divided into three groups: blank, casein and fibrin gel. The dorsal side of the rabbit ears was depilated with electric clippers and disinfected with povidone iodine, followed by 75% alcohol. The central artery of the rabbit ear was then incised with a sterile scalpel. After the wound bleeds freely for 5 s, the auricular root artery was compressed for 10 s and then wiped. The wound surface was immediately covered with the corresponding hemostatic material mentioned above, and absorbent cotton was used to absorb the exuded blood. Casein hydrogel bioadhesives formed *in-situ* for 30 s using an ordinary bright flashlight. Blood loss was calculated as follows: W = W_1_-W_0_, where W_1_ was the total weight of the material after hemostasis.

### ***In vivo*wound healing performance of hydrogel bioadhesive**

Male C57BL/6 N mice as above (8 weeks, 20 ± 2.5 g) were used and randomly assigned into 3 groups: (1) blank; (2) Casein; (3) Fibrin gel. After anesthesia as above, the dorsal hair was removed and the area disinfected, an 8 mm circular wound was created on the middle of each mouse’s back, and then different types of hydrogel precursor solution were added to fill the wounds and crosslinked *in-situ*, while no treatment was applied to the blank group. Casein hydrogel bioadhesives formed in-situ for 10 s using an ordinary bright flashlight. Hydrogel was left in-situ until degraded. The mice were sacrificed and the wound tissues were collected at predetermined time points for histological examination and assessment of inflammation. Meanwhile, the wound area was photographed and measured using ImageJ software.

The commercial enzyme linked immunosorbent assay (ELISA) kits (FANKEWEI, China) were used to assess inflammation in tissues from the wound area on the 4th day following manufacturer’s instructions.

### Histological analyses

The wound tissues were fixed in 4% paraformaldehyde, embedded in paraffin, and sectioned to 5 μm slides. After de-paraffin, hematoxylin and eosin staining was performed for histological analysis, and Masson staining (Solarbio, G1340, China) was performed for collagen quantification. Images were captured by a microscope (Leica DM3000, Germany). Collagen contents (*Collagen%*) were quantified by analyzing the proportion of aniline blue stained area (*S*_*Blue*_) in total tissue area (*S*_*Tissue*_) as the equation below. Pixel areas of *S*_*Blue*_ and *S*_*Tissue*_ were segregated from original images with function *Color Threshold* of ImageJ software.

$$Collagen\%=\frac{{S}_{Blue}}{{S}_{Tissue}}\times 100\%$$

### Immunofluorescent staining

Collected tissues were fixed with paraformaldehyde overnight. After dehydration with 40% sucrose buffer, tissues were embedded and 6 μm sections were made with a cryostat (Leica CM1950, Germany). The sections were washed with PBS, blocked with 5% BSA, and incubated with CD206 (1:100, AF2535, R&D systems), iNOS (1:200, 80,517, Proteintech), CD31 (1:00, AF, AF3628, R&D systems), α-SMA (1:300, 19,245, Cell Signaling Technology) at 4 °C overnight. After incubating with secondary antibodies (Abcam) and DAPI (Sigma), images were taken with a fluorescent microscope (Leica DM6B, Germany) and analyzed with ImageJ software.

### Statistical analysis

For each experiment, at least three samples were tested, and data were presented as means ± SD (*P < 0.05, **P < 0.01, ***P < 0.001, and ****P < 0.0001). All data obtained were in accordance with normal distribution with Shapiro-Wilk test. One-way analysis of variance (ANOVA) test and t-test were performed for statistical analysis (GraphPad Prism 8.2.1).

## Results

### Preparation and characterization of casein hydrogels

**Fig. 3:**
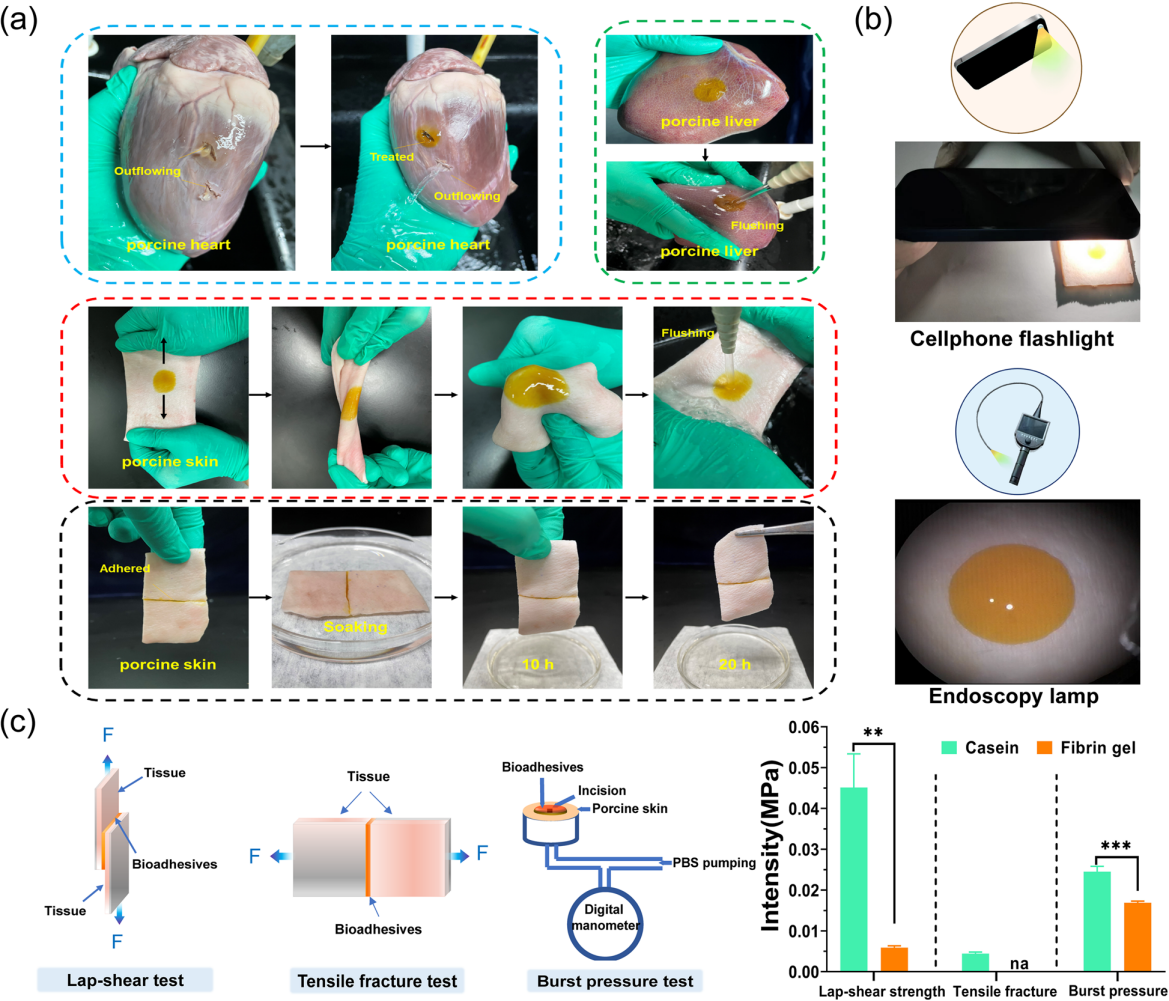
(**a**) Quick adhesion of casein hydrogel bioadhesives gelling *in-situ* in porcine heart, liver and skin, (**b**) Casein hydrogel photographs under cellphone flight and endoscopy lamp, (**c**) The lap-shear testing, tensile fracture testing and testing of adhesion performance with casein hydrogel bioadhesive and fibrin gel gelling *in-situ* on porcine skin (*n* = 3)

Figure 1a shows the white light photocrosslinking (Ru/SPS) mechanism to generate casein hydrogels through di-tyrosine bonds formation. Specifically, Ru^2+^ is photolyzed and excited to the transition state Ru^3+^ by the oxidant SPS and white light. Ru^3+^ having an oxidizing ability oxidizes tyrosine residues in protein molecules to form di-tyrosine bonds with other nearby tyrosine groups. At the same time, Ru^3+^ is reduced to Ru^2+^ to enter the cycle. The highly reactive tyrosine radicals attack other tyrosine residues to form di-tyrosine bonds, thereby forming protein chain crosslink [32]. The microstructures of different hydrogels were observed by scanning electron microscope (SEM) (Fig. 3a); the hydrogels with 10% and 15% casein concentration showed dense pores while 5% casein hydrogel showed a loose porous structure. The pore size decreased with the concentration of casein increasing, as the pore size of 5%, 10%, 15% casein hydrogels were 125.3 ± 84.4 μm, 49.5 ± 23.8 μm, 21.4 ± 15.4 μm, respectively (Fig. 3b). It is possible that the high-concentration polymer solution has high solid content and high-density tyrosine residues, and then forms a high-density network structure after gelation.

The FTIR spectra demonstrate the chemical changes in the secondary structure of the original casein powder and photocrosslinked casein hydrogel samples. From the FTIR results in Fig. 3c, the intensity of peak at 1136 cm^− 1^, which were assigned to stretching vibration of the di-tyrosine C − C bond, increased in the spectrum of the casein hydrogel. The formation of the di-tyrosine C − C bond was also confirmed by the solid-state NMR results. The resonance at 117 ppm, which was assigned to the phenol carbon of tyrosine residues, shifted to a lower field at 120 ppm due to the di-tyrosine cross-link (Supplementary Fig. 1a). A shift from 245 nm to 237 nm of the peak in CD spectra also indicated a structural change of tyrosine residues in casein (Supplementary Fig. 1b). From the XRD profiles, both casein powder and casein hydrogel showed the same peaks at the 2theta of 9° and 20°, indicated that the crystalline state of the casein did not change after photocrosslinking (Supplementary Fig. 1c). Hence, the di-tyrosine cross-link was produced in the casein with the Ru/SPS redox system.

The rheological properties of casein hydrogel were monitored during the photo-curing process to evaluate the effect of Ru, SPS and casein concentration on the gelation time and storage modulus (G′). After exposure to visible light, the storage modulus begins to increase to a certain point until it gradually plateaus. Increasing Ru (0.2 mM ~ 2 mM) and SPS (30 mM ~ 80 mM) concentration reduced gelation time of casein hydrogel, while the storage modulus of casein hydrogel was positively correlated with Ru and SPS concentration (Supplementary Fig. 2a, b). However, when Ru concentration was higher than 1mM, SPS concentration was higher than 40 mM, further increase of initiator concentration only shortened gelation time in a small scale (Supplementary Fig. 2b). To minimize the potential toxicity from the initiator, we used 40 mM SPS and 1mM Ru in the following experiments if not specifically indicated.

Figure 3d shows the effect of casein concentration on the gelation time, with 58.92 ± 1.35 s for 5% casein, 18.62 ± 1.26 s for 10% casein, and 34.76 ± 0.98 s for 15% casein. The final storage modulus after complete gelation was 2400.57 ± 121.34 Pa for 5% casein, 5875.51 ± 223.18 Pa for 10% casein, and 430.33 ± 101.69 Pa for 15% casein. 10% (w/v) casein concentration showed a much faster gelation and higher storage modulus compared to casein solutions with lower (5%) or higher (15%) concentrations (*P <* 0.0001) (Fig. 3d), so we used 10% casein for the rest of experiments if not specifically indicated. The intensity of light source had a great influence on the gelation rate, when increasing light intensity from 1.5 mW cm^− 2^ to 40.2 mW cm^− 2^ (with a wavelength of 450 nm), casein hydrogel can be solidified in 2.15 ± 0.41 s (Fig. 3e). In addition, white light exerted ultrafast gelation rate, when we used an outdoor flashlight or a cellphone flash as the rheological light source, and the gelation time of casein hydrogel was 4.53 ± 0.66 s and 18.39 ± 1.59 s, respectively (Fig. 3d).

In this system, the maximum absorption peak of the photoinitiator is around 450 nm. Therefore, common white light sources were able to initiate crosslinking and gelation of casein hydrogel. The distance and light intensity are negatively correlated (Supplementary Fig. 2c), and the gelation time increases as the light intensity decreases (Fig. 3e). In the following experiments, 1 cm distance from the lamp was chosen for ease of operation and speed of gelation. Considering the different light intensity of different light sources and the time needed for fully gelation, we used an exposure time of 3 min to get stable results.

We characterized the mechanical properties of different hydrogels by studying the stress strain curves of compression and elongation (Supplementary Fig. 2d and 2e). The compression stress at break of the 10% (w/v) casein hydrogel (45.33 ± 0.58 kPa) was significantly higher than that of other hydrogels (5.03 ± 1.27 kPa of 5% casein and 22.61 ± 0.85 kPa of 15% casein) (Fig. 3f), which was consistent with the storage modulus results in the rheological experiments (Fig. 3d). The compression stress at break increased according to the SPS concentration, which was consistent with the storage modulus results in the rheological experiments (Supplementary Fig. 2e).

The swelling of hydrogels is ubiquitous but disadvantageous in biomedical applications such as tissue engineering and internal wound closure [33]. The swelling of hydrogels not only deteriorates the mechanical properties of hydrogels, but also oppresses the surrounding tissues when used in confined spaces [34]. The swelling ratio was measured at different time points after soaking the samples in PBS for 168 h (Fig. 3 g). Especially in the soaking process, the 10% casein hydrogel showed no obvious swelling, while the 5% casein hydrogel showed a decrease and 15% casein hydrogel showed an increase in volume and weight. The observed change of hydrogel swelling rate with different casein concentrations may due to the variation of molecular chain interlacing and osmotic pressure along with the casein concentration [35]. Casein is a mixed protein composed of hydrophobic section and hydrophilic sections, which may lead to micelle-like structure in water [36]. The dissolving process in alkaline condition help to the micelle dissociate and molecule chain interpenetrate under stirring. In 5% casein solution, although low concentration reduces the probability of contacts between tyrosine groups, which resulted in large pore size in SEM images, the molecule chains are fully interpenetrated and the reactions between tyrosine groups are mainly intermolecular. The shrink of hydrophobic sections when soaking in PBS may lead to the reduce of mesh size within hydrogel, which resulted in a negative swelling ratio of the 5% casein hydrogel. This hypothesis was supported by the pore size decrease in SEM images after soaking in PBS for 168 h (Supplementary Fig. 3). For casein solution with higher concentrations, the molecular chains become condensed, resulting in insufficient chain extension, thus the coupling within the same chain may increase during gelation process [37]. In 15% casein, although the high concentration resulted in higher amount of di-tyrosine coupling, the inadequate intermolecular crosslinking can result in network relaxation of the hydrophilic section of the protein chains in swelling experiment. In 10% casein, the degree of cross-linking was the highest and the chain condensation and osmotic pressure reach a balance, resulted in a low swelling ratio, thus we used 10% casein for the rest of functional studies.

To test the stability of casein hydrogel, rheological analysis was used to explore the stability of casein hydrogel precursor solution over storage and freeze-thaw process (Supplementary Fig. 4). The results showed that cryopreservation (-20 °C) for 21 days and freezing-thawing for 6 times had no significant influence on gelation time and storage modulus of casein hydrogel. However, refrigerating (4 °C) for 7 days prolonged the gelation time and reduced the storage modulus, which may due to the partial degradation of casein during refrigeration.

To demonstrate the possibility to build up the complex architecture from photocrosslinkable casein, 3D printing was proceeded on a digital light processing printer. As shown in the Fig. 3 h, the 10% (w/v) casein hydrogel precursor solution was successfully crosslinked layer by layer into snowflake, mesh dressings and eagle patterns, showing good shape fidelity.

### Tissue adhesion of casein hydrogel bioadhesives

To demonstrate the potential applications of the casein hydrogel as bioadhesive, we next evaluated adhesion of the casein hydrogel bioadhesives in vitro (Fig. 2a). Casein itself has certain viscidity in nature which has been used as adhesive [38]. We found that casein hydrogel can adhere to dry surface such as paper (Supplementary Movie 1), however, it cannot adhere to wet tissue surface (Supplementary Movie 1). We speculated that the formation of di-tyrosine bonds between tyrosine residues in the casein hydrogel and tyrosine residues on tissue surface initiated by photocrosslinking was responsible for the tissue adhesive effect. As a result, we applied casein hydrogel precursor solution and photocrosslinked *in-situ* to induce tissue adhesion. With *in-situ* gelation, the casein hydrogel bioadhesive could seal damaged porcine heart (5-mm-long strip wound) (Supplementary Movie 2). Moreover, the casein hydrogel precursor solution could also form stable adhesion on porcine skin and liver after *in-situ* white-light initiated gelation. The casein hydrogel bioadhesives strongly adhered to these tissues despite distorting, bending, or water flushing. Furthermore, the casein hydrogel precursor solution dripped between two pieces of porcine skin could form hydrogel bioadhesive *in-situ*, which enabled strong adhesion even after soaking in PBS for 20 h. Two pieces of porcine skin soaking in PBS was successfully bonded using casein hydrogel bioadhesive with an outdoor flashlight (Supplementary Movie 3). Gelation was initiated with a cellphone flashlight, or an endoscopy lamp within 30–60 s (Fig. 2b). This indicated the formation of chemical bonding between casein hydrogel and tissues, which is the formation of di-tyrosine bonds.

To quantitatively assess the adhesive strength of the casein hydrogel bioadhesives, we performed a lap-shear test (Fig. 2c). The adhesive strength of the casein hydrogel bioadhesive was 45.13 ± 5.63 kPa, which was significantly higher than that of commercially available surgical fibrin gel (5.90 ± 0.53 kPa; *P <* 0.01). We then performed the tensile fracture tests to further characterize the adhesive property of the casein hydrogel bioadhesive (Fig. 2c). The tensile fracture strength of casein hydrogel bioadhesive was 4.47 ± 0.23 kPa, whereas fibrin gel failed in the test due to a lack of sufficient mechanical strength to support the operation for mechanical test (Supplementary Movie 4, 5). A burst pressure test was used to evaluate the capacity of the casein hydrogel when resisting the peeling force from the tissue while rapidly adhering to the biological surface. The measured burst pressure of the photocrosslinked casein hydrogel bioadhesive was 24.50 ± 0.85 kPa (184.21 ± 6.39 mmHg), which was higher than that of fibrin gel (16.87 ± 0.34 kPa, 126.84 ± 2.56 mmHg), as shown in Fig. 2c.

### Cytotoxicity and biodegradability

**Fig. 4:**
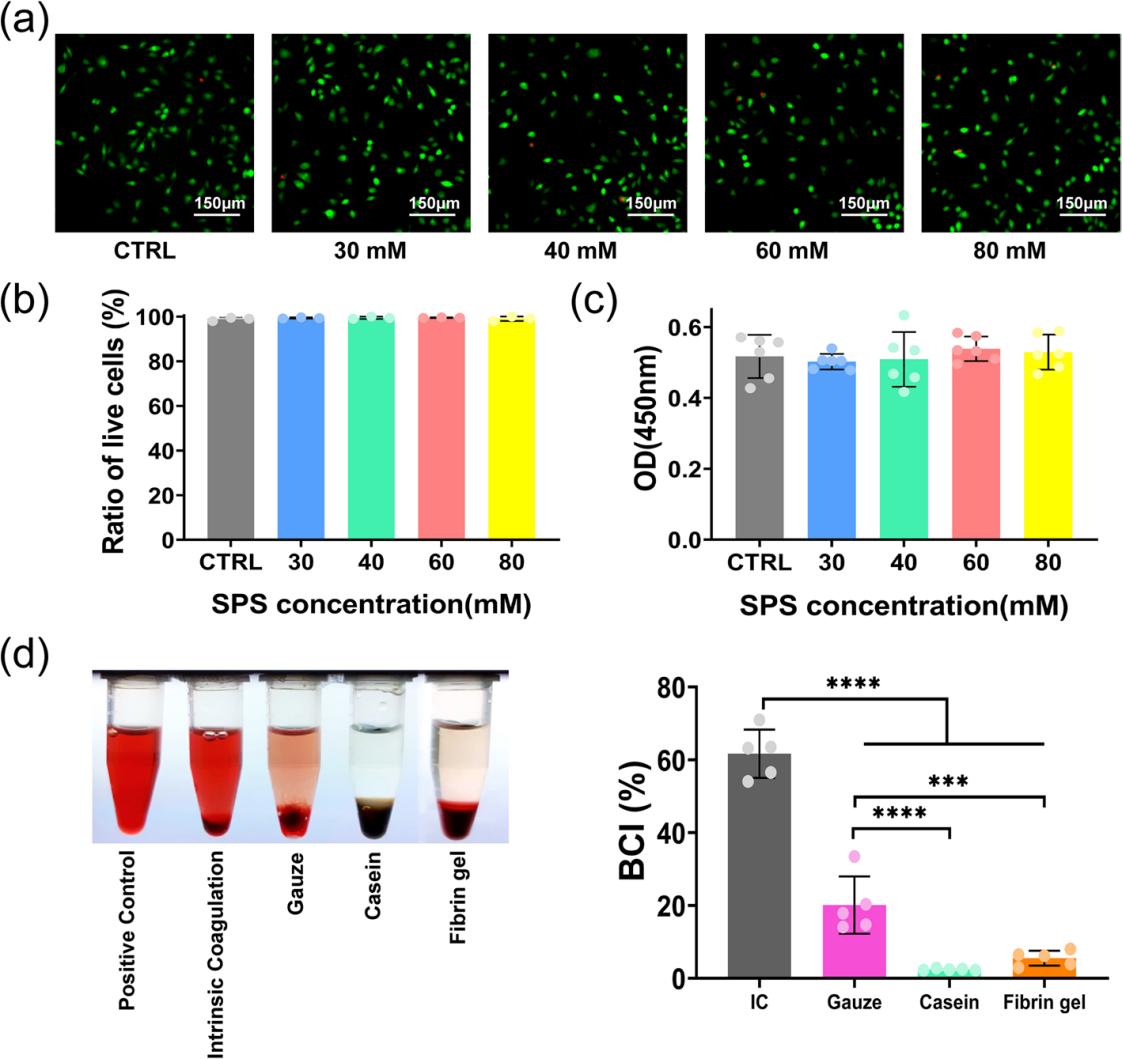
Cytocompatibility of casein hydrogels. (a) Fluorescent microscope images of live/dead staining of L929 after exposed to 24 h leaching solution of casein hydrogels produced with varying SPS concentrations (30, 40, 60, 80 mM); (b) The ratio of live cells (*n* = 3) and (c) the OD value at 450 nm of CCK-8 assay for cells treated with casein hydrogel extracts produced with different SPS concentrations (*n* = 5); (d) In vitro coagulation of casein hydrogel bioadhesives (*n* = 5)

To prove the applicability of casein hydrogel for biomedical applications, the cytotoxicity was investigated against L929 mice fibroblast. As shown in Fig. 4a - c, the cell viability in hydrogel extracts was similar to the blank group. Besides, there was no difference in cell viability of casein hydrogel prepared with different SPS concentration, indicating that these hydrogels have good cell compatibility. In addition, 35 days after implantation, bulk casein hydrogel basically degraded, demonstrating casein hydrogel has good biodegradability (Supplementary Fig. 5).

### In vitro and in vivo hemostatic performances

In vitro blood clotting index (BCI) is a common test method to evaluate the ability of hydrogel bio-adhesive coagulation and hemostasis [39]. The lower BCI indicates higher coagulation efficiency. Gauze was defined as the hemostatic control group and natural coagulation was selected as blank control group. After 1 min incubation with different dressings and blood at 37℃, the BCI values of each bioadhesive were lower than those with natural coagulation and gauze (Fig. 4d). In addition, the BCI of casein hydrogel bioadhesive was lower than that of fibrin gel. Thus, photocrosslinked casein hydrogel bioadhesive has higher hemostatic capacity than gauze and fibrin gel.

**Fig. 5:**
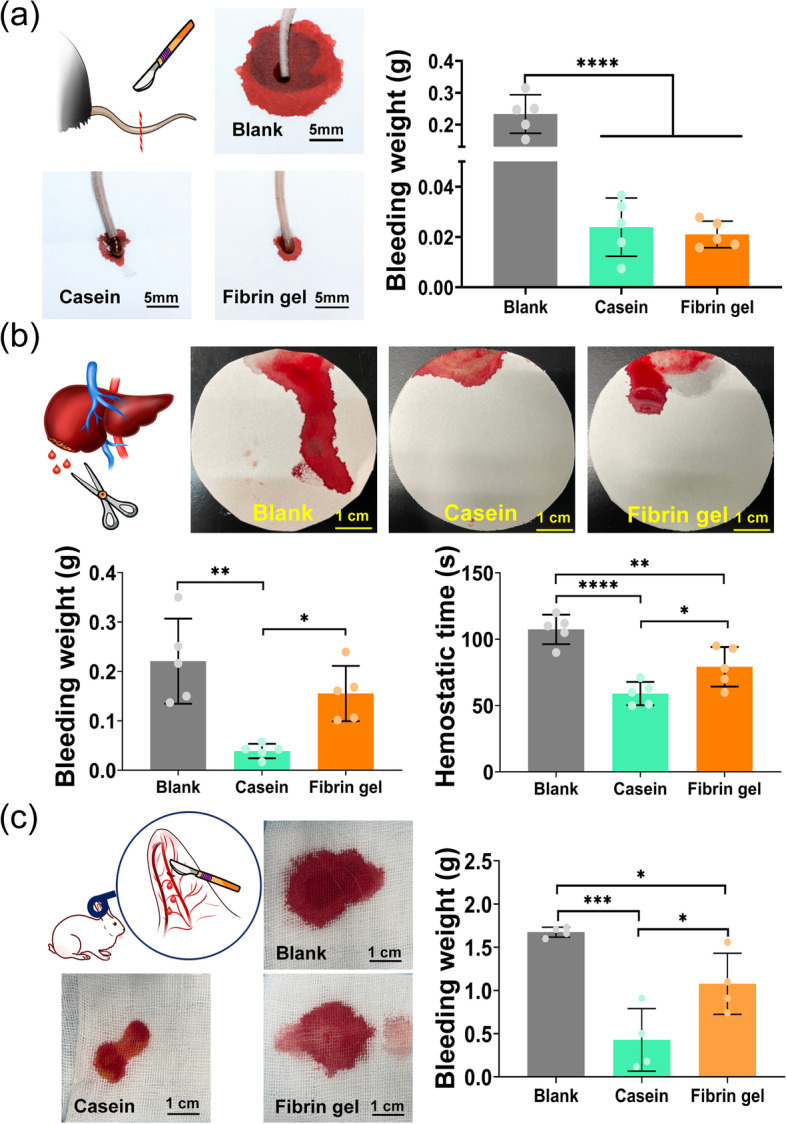
In vivo hemostasis tests of casein hydrogel bioadhesives. (**a**) Bloodstain photographs and quantitative results of blood loss in a mouse-tail amputation model (*n* = 5); (**b**) Bloodstain photographs and quantitative results of blood loss in the mouse liver hemorrhage model, scale bar:1 cm (*n* = 5). (**c**) Bloodstain photographs and quantitative results of blood loss in ear artery hemorrhage model of rabbits, scale bar:1 cm (*n* = 4)

The hemostatic performance of the casein hydrogel bioadhesive was also tested in mice-tail amputation model, mice-liver injury model and rabbit-ear artery hemorrhage model. In the mice-tail amputation model, compared with the blank group (233.26 ± 81.83 mg), the blood loss in the casein hydrogel bioadhesive group was significantly reduced (23.93 ± 12.76 mg) (*P* < 0.0001) (Fig. 5a). It is worth mentioning that there was no significant difference in blood loss between casein hydrogel bioadhesive and fibrin gel (21.13 ± 10.86 mg). However, in a liver hemorrhage model with 1/3 left lobe excision, the injection of the casein hydrogel bioadhesive and white light irradiation resulted in quick hemostasis (59.0 ± 9.3 s) and minimum amount of blood loss (38.8 ± 18.4 mg) (Supplementary Movie 6, Fig. 5b). Meanwhile, the blank control showed large amount of blood loss (220.8 ± 90.6 mg) and longer bleeding time (107.4 ± 16.6 s) (Supplementary Movie 7, Fig. 5b). As a positive control, commercialized hemostatic gel -- fibrin gel had a blood loss of 55.3 ± 53.8 mg and a hemostatic time of 79.2 ± 16.2 s, both were significantly higher than casein hydrogel bioadhesive (Supplementary Movie 8, Fig. 5b). In vivo hemostatic efficiency was further evaluated by an ear artery hemorrhage model of rabbits (Fig. 5c). Blood loss in the casein hydrogel bioadhesive group (0.43 ± 0.31 g) was much lower than the blank group (1.68 ± 0.06 g), and fibrin gel group (1.08 ± 0.48 g) (Supplementary Movies 9, 10 and 11, Fig. 5c). In addition, a rat abdominal aorta injury model and a porcine skin injury model were added to evaluate the hemostatic properties of the casein hydrogel in massive hemorrhage or large animals. Compared with the blank group, the casein hydrogel effectively reduced bleeding in both of the models (Supplementary Movies 12,13,14 and 15, Supplementary Fig. 6).

The hemostatic effect of casein hydrogel bioadhesive *in-situ* was mainly due to its rapid photocrosslinking response and the wet tissue adhesion ability, which facilitated the adherence to the wound and provided a stable gel network as a physical barrier to accelerate blood coagulation.

### Wound healing efficiency

**Fig. 6:**
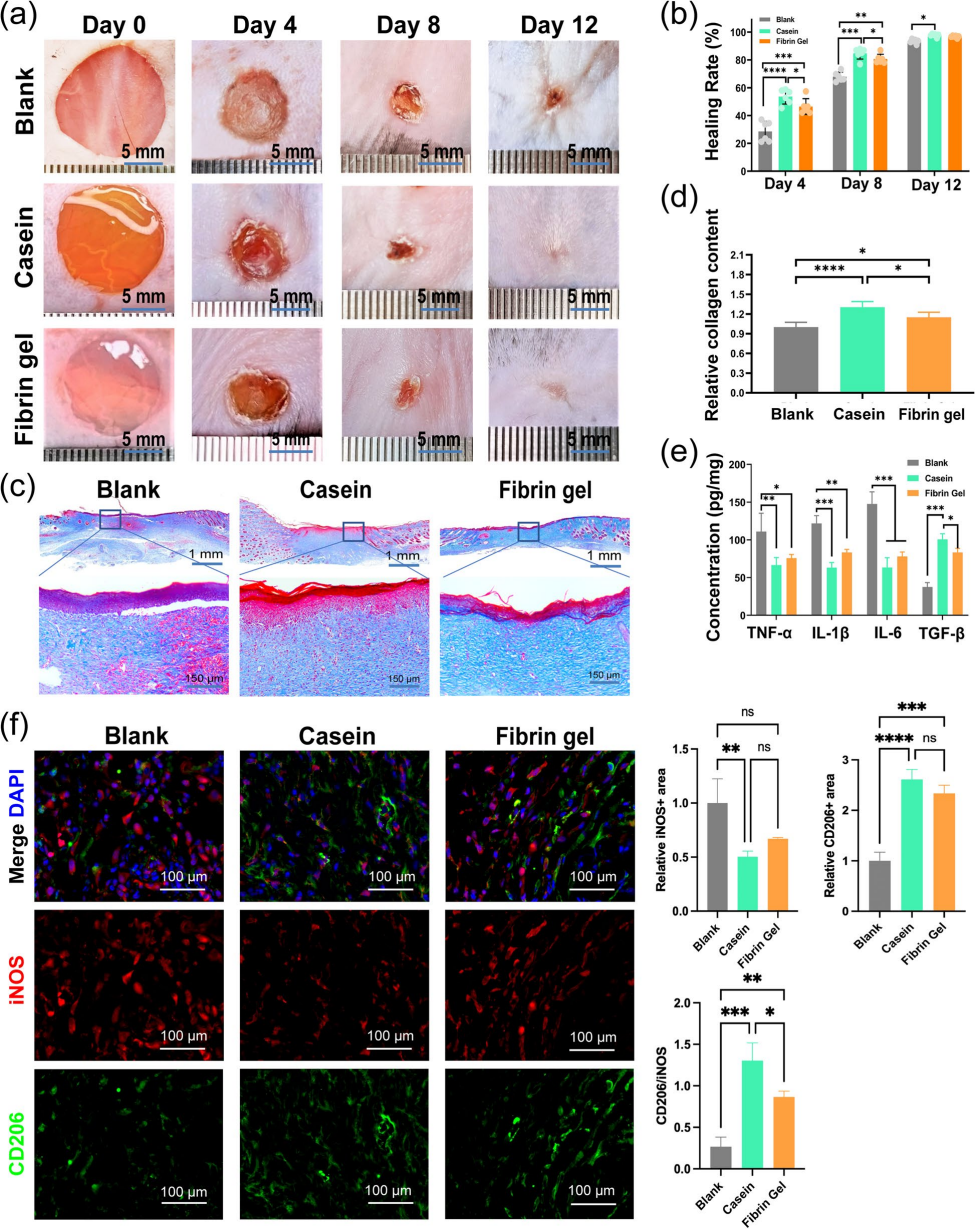
Evaluation of casein hydrogel bioadhesive in promoting wound repair of mice dorsal skin; (**a**) Images of the wound healing site with different treatment groups at day 4, day 8 and day 12 ; (**b**) Statistical data of wound closure ratio with different treatment groups (*n* = 6); (**c**) Masson staining of wound tissues at day 12; (**d**) Quantitative counting of collagen fiber staining (*n* = 6); (**e**) The expression intensity of inflammation related chemokines in full-thickness wound tissues with different treatments at day 4 were extracted and evaluated by ELISA (*n* = 3); (**f**) Immunofluorescence staining results of wound regeneration site with different treatment groups at day 4 with iNOS and CD206

The above experimental results show that casein hydrogel bioadhesive has suitable mechanical strength, adequate tissue adhesiveness, good hemostatic ability and good cytocompatibility. Thus, it can be used as powerful wound dressing to improve wound healing. As a promising method for the preparation of hydrogels, white light-induced crosslinking can control the reaction kinetics in time and space and polymerize rapidly under mild conditions [40]. The wound closure efficacy of hydrogel bioadhesives was determined in vivo by creating full-thickness dorsal skin incisions in laboratory mice (C57BL/6 N), and then applying hydrogel bioadhesives to the wound site. Figure 6a shows the images of the wound taken at different time intervals without treatment or with different hydrogel bioadhesives. The growth of the new epidermis extended to the wound center with all treatment conditions, thereby reducing wound area. Scabs were formed and fell off before healing in all groups. To demonstrate the fate of casein hydrogel more clearly, fluorescently-labeled casein hydrogel was gelled in situ at the wound, and the state of the hydrogel was recorded by fluorescence stereomicroscope at different time points (Supplementary Fig. 7). The fluorescently-labeled hydrogel fell off on about the 6th day. Among different groups, the casein hydrogel bioadhesive showed the most accelerated wound contraction in the 12-day period compared to that with blank and fibrin gel, indicating that treating with casein hydrogel bioadhesives improved and accelerated wound healing.

Moreover, 4 days post-transplantation, the closure percentage of the wounds treated with casein hydrogel bioadhesives was approximately 53.5% (*P* < 0.01), whereas wounds treated with blank and fibrin gel showed lower wound closure percentage (28.6% and 46.4%, respectively; Fig. 6b). To account for this phenomenon, we performed histological evaluation and assessment of inflammation around the wounds at day 4 (Supplementary Fig. 8a). All groups had granulation tissues filling the wound up at day 4, whereas the blank group arrested in a relatively immature stage, with scattered blood clots. Among the hydrogel administered groups, the regenerated tissues of casein hydrogel treated group exhibited a phase of early fibrosis at the contact surface, indicating casein hydrogel facilitated early healing of wound tissues.

Sections taken on day 12 demonstrated mature epidermal regeneration in the hydrogel groups, while the blank group had not formed a complete structure of healed pattern. In wound healing, the amount of regularly deposited collagen is proportional to the strength of healed tissue [41]. Thus, we performed Masson staining to visualize the morphology of collagen after a 12 days healing phase. The collagen was stained blue, while cells and other components were stained pink. Both hydrogel groups demonstrated healthier healings, with less residual inflammation and more deposited collagen content in dermis layer (Fig. 6c). A quantitative counting of fibers demonstrated the highest amount of collagen deposition in casein treated group with 1.31 ± 0.09 folds in collagen density compared to blank group (Fig. 6d). To further study the wound healing effect, we analyzed the myofibroblast differentiation gene expression, the inflammation, and the vascularization of healing tissues. As the related cytokines or genes mostly take an important role at early stage [42], we took qPCR (Supplementary Fig. 8b) and ELISA (Fig. 6e) analysis of the wound-healing tissues at day 4. It has been recognized that *in-situ* TGF-β, which ameliorates inflammation and activates myofibroblasts, is necessary for granulation stage in wound healing [43]. Compared to untreated group, we found casein treated wounds expressed a higher level of TGF-β (Fig. 6e), and thus a higher level of α-SMA and Col1a1 (Supplementary Fig. 8b), which marks active myofibroblasts.

To further study the vessel cell activation, which is considered to support the regenerative activities [44], we took IF staining of CD31 and α-SMA to visualize the small vessels in wound healing tissues, which indicated a higher density in casein treated tissues. In consistence with IF results, we demonstrated a higher expression of VEGFa in casein treated wounds with qPCR analysis (Supplementary Fig. 8c). However, no significant difference was found between casein group and fibrin group.

On the other side, prolonged high levels of inflammatory cytokines cast a detrimental effect [45]. As a result, we found a lower inflammation level of TNF-α, IL-1β, IL-6 in casein group compared to untreated group, with both ELISA (Fig. 6e) and qPCR (Supplementary Fig. 8b). However, no significant difference was found between hydrogel groups. Moreover, we preformed immunofluorescence (IF) staining of iNOS and CD206 to check the phenotype of immune cells. By counting the density of iNOS+ (M1 marker) and CD206+ (M2 marker) immunocytes, we found the highest CD206+/ iNOS + was shown in casein group (1.30 ± 0.21), significantly higher than 0.27 ± 0.12 in blank group and 0.87 ± 0.07 in fibrin gel group (Fig. 6f). This indicated that application of casein hydrogel promoted differentiation of macrophages towards M2 phenotype, rather than inflammation-accelerating M1 type [46].

In general, our results demonstrated that *in-situ* application of casein hydrogel resulted in a better repairing property and stronger strength of the regenerated tissue in wound healing.

## Discussion

Casein, as a protein from food source, accounting for about 80% of the proteins in bovine milk [47], is more low-cost and abundant compared to other naturally derived proteins, such as collagen, gelatin, silk, keratin, elastin, and fibrin. This research work pioneered developed a multifunctional casein hydrogel formed by photo-crosslinking via white-light initiation for biomedical application. We optimized the composition of the reaction system to achieve fast gelation, high mechanical strength and high biocompatibility. In single-factor experiments, the concentration of casein, Ru, and SPS were optimized with gelation time and mechanical properties of casein hydrogels. Overall, the content of tyrosine residue in protein has a great influence on the properties of the formed hydrogel; Sufficient Ru and SPS concentration could accelerate the formation of cross-linked chains and increase the degree of cross-linking. The abnormal gelation time and modulus variation when the concentration of casein solution increased from 10 to 15% might due to the slow molecule diffusion in highly concentrated casein solutions, which hindered the contact between tyrosine residues.

Based on the fast gelation speed and comparable mechanical strength of casein hydrogels, casein hydrogel can be used as raw materials for 3D printing to generate complex structures. The key printing parameters, including printing speed, light intensity and solution temperature, were similar to those used in popular biological inks such as silk fibroin [22] and gelatin [20]. Compared with gelatin with certain condensation properties, the casein biological ink had a wider range of temperature adaptability. Hence, the casein hydrogel precursor solution as potent bio-ink is expected to be manufactured into personalized complex hydrogel scaffolds.

Casein hydrogels were formed rapidly in white-light-induced catalysis. The casein hydrogels were all induced to crosslink when exposed to flashlight, cellphone flash, and endoscope, indicating that casein hydrogels have strong applicability for daily life as well as medical, and military tool (Supplementary Movie 16 and 17). The fast polymerization characteristics allowed them to rapidly and effectively seal wounded tissues. The gelation mechanism through di-tyrosine crosslinking of casein conferred strong adhesion between casein hydrogel bioadhesive and tissue. The adhesive strength in both lap-shear and tensile fracture test presented much higher values than the commercial fibrin bioadhesives. In addition, the burst pressure of casein hydrogel bioadhesive is also significantly higher than that of gelatin hydrogel bioadhesive with the Ru used as a photoinitiator in a previous study [40] and blood pressure in most clinical settings (systolic BP 7.98–21.28 kPa) [48]. In the burst pressure test, the casein hydrogel bioadhesive was raptured just above the puncture hole without separation from the porcine skin tissue. This indicates that the tissue adhesion of the casein hydrogel bioadhesive is higher than its mechanical strength, which can form strong bonds with the tissue substrate.

In trauma caused by accidences and surgery, certain amounts of bleeding are to be expected, so hemostasis is required for wound treatment [49]. The ideal wound dressing should have the ability to clot blood to accelerate hemostasis [50]. Casein hydrogel bioadhesive had the best hemostatic properties in the mouse liver injury model, manifesting the shortest hemostasis time and the lowest bleeding volume. During the treatment process, the casein hydrogel bioadhesive could rapidly gel *in-situ* and adhere to the moist wound surface to seal the liver incision injury under white light irradiation. while fibrin gel was washed away by blood flow, hindering its hemostatic properties, which was consistent with previous study reports [5, 48]. The rapid wound sealing and strong adhesion of casein hydrogel bioadhesives may be the main factors for the excellent hemostatic properties, enabling casein a promising candidate for wound hemostasis. The most commonly used photocrosslinking catalysator requires UV light to be activated. However, UV radiation may damage cellular DNA and cause cells to generate reactive oxygen species (ROS), which can lead to cytotoxicity [51]. Previous studies have shown that cells generate negligible amounts of ROS when exposed to visible light (wavelength: 455 nm, 30 mW/cm^2^ for 30 s) [40]. Due to the high optical extinction coefficient of blood and blood-containing tissue lies in < 420 nm blue spectral region, the photoinitiator at the corresponding excitation wavelength cannot be effectively activated, which affects the gelation efficiency [52]. It has been previously reported that modified natural materials have the ability of wet wound adhesion and rapid gelation [53–56]. However, the modifications processes are relatively complicated, and a large amount of hazard molecules such as methacrylic acid [40], N-(2-aminoethyl)-4-(4-(hydroxymethyl)-2-methoxy-5-nitrosophenoxy) butanamide [57, 58], periodate [59] were applied during preparing course, which may lead to a certain impact on the environment. Surprisingly, our cross-linking reaction did not require any pretreatment of the protein, so it may be extended to other proteins.

Apart from quick cross-linking and great adhesiveness to preserve wound tissues in the first place, the casein hydrogel bioadhesive demonstrated better wound healing efficiency in a mouse full-thickness cutaneous wound model compared to fibrin gel in terms of wound closure percentage pathological pattern. This may be attributed to a better tissue affinity of casein hydrogel to attach well and sustain a suitable microenvironment for tissue regeneration. For the early stages of healing, it is generally believed that TGF-β act as a stimulator for cell proliferation and differentiation to maintain homeostasis and maturation of granulation tissues [60], while inflammatory factors, such as TNF-α, IL-1β, IL-6 hinder the wound healing and take the responsibility for adverse remodeling [61, 62]. Our results revealed that casein hydrogel reduced those inflammatory factors and upregulated TGF-β level at granulation stage, which is ideal for wound healing. Moreover, we investigated the terminal stage of healed area, and thus showed that regenerated skins deposited more arranged collagen with preservation of casein hydrogel, indicating a stronger mechanical strength of healed tissue [41].

## Conclusion

The work presents a novel milk derived first-aid bioadhesive which can crosslink and covalently bind to tissues under more clinically relevant white-light radiation in 2 s. The strong dressing facilitated rapid hemostasis in visceral hemorrhage (liver and abdominal aorta) in rodent models and body surface bleeding for larger animals. The great biocompatibility of the casein hydrogel bioadhesive facilitated great wound healing effects compared to the commercial fibrin gel dressing in a mouse model of full thickness skin defect. With these attractive properties, we firmly believe that the white-light crosslinkable casein bioadhesive has great potential to be translational as a powerful bioadhesive for hemostasis in emergency wound management.

## Supplementary Information

**Additional file 1: Supplementary Material 1.**

**Additional file 2: Supplementary Movie 1.**

**Additional file 3: Supplementary Movie 2.**

**Additional file 4: Supplementary Movie 3.**

**Additional file 5: Supplementary Movie 4.**

**Additional file 6: Supplementary Movie 5.**

**Additional file 7: Supplementary Movie 6.**

**Additional file 8: Supplementary Movie 7.**

**Additional file 9: Supplementary Movie 8.**

**Additional file 10: Supplementary Movie 9.**

**Additional file 11: Supplementary Movie 10.**

**Additional file 12: Supplementary Movie 11.**

**Additional file 12: Supplementary Movie 12.**

**Additional file 13: Supplementary Movie 13.**

**Additional file 14: Supplementary Movie 14.**

**Additional file 15: Supplementary Movie 15.**

**Additional file 16: Supplementary Movie 16.**

**Additional file 17: Supplementary Movie 17.**

## Data Availability

The datasets used and/or analyzed during the current study are available from the corresponding author on reasonable request.
