# Supplementary material for: White-light crosslinkable milk protein bioadhesive with ultrafast gelation for first-aid wound treatment
Source: Biomater Res. 2023 Feb 3;27:6. doi: 10.1186/s40824-023-00346-1 (PMC9898936; doi:10.1186/s40824-023-00346-1)
Supplement: Supplementary file 1 — Additional file 1: Supplementary Material 1. [file 40824_2023_346_MOESM1_ESM.docx]

Supporting Information

White-light crosslinkable milk protein bioadhesive with ultrafast gelation for first-aid wound treatment

Qinchao Zhu^1,†^, Xuhao Zhou^2,†^, Yanan Zhang^3^, Di Ye^4^, Kang Yu^5^, Wangbei Cao^6^, Liwen Zhang^6^, Houwei Zheng^6^, Ziyang Sun^7^, Chengchen Guo^7^, Xiaoqian Hong^2^, Yang Zhu^6^, Yajun Zhang^8^, Ying Xiao^8^, Teresa G. Valencak^1^, Tanchen Ren^2,*^; Daxi Ren^1,*^

^1^ *Institute of Dairy Science, College of Animal Sciences, Zhejiang University, Hangzhou,310058, China*

^2^ *Department of Cardiology, Cardiovascular Key Laboratory of Zhejiang Province, Second Affiliated Hospital, School of Medicine, Zhejiang University, Hangzhou, 310027, China*

^3^ *Key Laboratory of Animal Virology of Ministry of Agriculture, Center for Veterinary Sciences, Zhejiang University, Hangzhou, 310058, China*

^4^ *Department of Veterinary Medicine, College of Animal Sciences, Zhejiang University, Hangzhou, 310058, China*

^5^ *Key Laboratory of 3D Printing Process and Equipment of Zhejiang Province, School of Mechanical Engineering, Zhejiang University, Hangzhou 310027, China*

^6^ *MOE Key Laboratory of Macromolecular Synthesis and Functionalization, Department of Polymer Science and Engineering, Zhejiang University, Hangzhou 310027, China*

^7^ *School of Engineering, Westlake University, Hangzhou, Zhejiang, 310023, China*

^8^ *Sir Run Run Shaw Hospital, School of Medicine, Zhejiang University, Hangzhou 310020, Zhejiang, China*

†Qinchao Zhu and Xuhao Zhou contributed equally to this work

*Corresponding Authors：

Daxi Ren: [dxren@zju.edu.cn](mailto:dxren@zju.edu.cn), Tanchen Ren: rentanchen120@zju.edu.cn .

**Method and material**

**Solid-state nuclear magnetic resonance (ssNMR)**

The ssNMR experiments were performed on Bruker 500 MHz spectrometer (Bruker, Germany) equipped with a 3.2 mm triple resonance probe. 1H→13C cross-polarization magic angle spinning (CP-MAS) NMR spectra were obtained with the CP condition of a 2.77 μs 1H π/2 pulse with a power of 95 W, followed by a 2 ms contact time at a power of 84.2 W. The experiments were conducted at 2.0s recycle delay, 37 kHz sweep width, 2048 scans, and the magic angle spinning speed of 20 kHz.

**Circular dichroism (CD) spectroscopy**

Circular dichroism (CD) spectra were collected on Chirascan V100 with spectrum range of 185-260 nm with an integrating sphere attachment. For each test, average was taken from three measurements.

**X-Ray diffraction (XRD)**

The XRD profiles were collected on an X-ray Diffractometer (Bruker, D8 Advance, Karlsruhe, Germany). Each sample was characterized with a tube voltage of 40 kV, a current of 40 mA, and a plate rotational speed of 5 rad/min. The wavelength (λ) of X-ray was 1.54 Å and the diffraction angle (2θ) was recorded from 5° to 60°.

**Rheology of casein hydrogel**

The dynamic storage (G′) and loss modulus (G″) of the above compositions were measured by the rheometer (MCR302, Anton Paar, Austria) equipped with a Peltier element for temperature control and a generator. The 10% w/v casein solutions with various Ru (0.2 – 2 mM) and SPS (20 Mm – 80 mM) concentrations were placed between the plates at 37 °C to completely fill the gap (0.5 mm). Under 1.5 mW cm^−2^, 450 nm visible irradiation, time sweep oscillatory measurements were performed at a frequency of 30 Hz and 1% strain. The point where G′ and G′′ intersect is considered to be the gelation point; while the point where the elastic modulus reaches a plateau is taken as the complete crosslinking.

**Mechanical test**

The hydrogel precursor solution with various casein (5% – 15%, w/v) and SPS (20 Mm – 80 mM) concentrations gelled in the same volume and shape (8 mm diameter and 6 mm height) after irradiated with a white LED lamp (10 W) from a distance of 1 cm for 3 min. The stress-strain curve was obtained via a mechanical tester (Instron 5543A) with a rate of 1 mm/min. The area underneath the curve is the toughness of the hydrogels. Three parallel samples were measured to calculate the average values.

***In vivo* degradation**

10% w/v casein hydrogel precursor solution were exposed to visible light in a 1 mL syringe to form cylinder hydrogels. Hydrogel samples were cut into 0.1 g pieces. Male C57BL/6N mice (20 g) were anesthetized with pentobarbital. After the hair on the back was removed and disinfected, the skin was incised symmetrically on both sides of the midline. The crosslinked hydrogels were implanted subcutaneously and the wound were sutured (N = 3 in each group). At 7 days, 21 days and 35days after implantation, the animals were sacrificed, the hydrogels and surrounding tissue were excised and photographed.

**mRNA expression in wound healing tissues**

Total RNA was isolated from tissues with RNA isolater reagent (R401, Vazyme) following the manufacturer’s instruction. cDNA was synthesized with HiScript RT SuperMix for qPCR (R122, Vazyme). Gene expression was performed by qPCR using SYBR Green (4309155, Invitrogen) and primer pairs (Table S1). 18S (B661301, Sangon Biotech) served as a reference gene. Data analysis was performed using the ∆∆Ct method.

**Table S1: Primers used in qPCR**

| **Genes** | **Primer sequences** |
| --- | --- |
| Col1a1 | **Forward:** 5’- TGAACGTGGTGTACAAGGTC -3’ |
|  | **Reverse:** 5’- CCATCTTTACCAGGAGAACCAT -3’ |
| VEGFa | **Forward:** 5’- TAGAGTACATCTTCAAGCCGTC -3’ |
|  | **Reverse:** 5’- CTTTCTTTGGTCTGCATTCACA -3’ |
| IL-1b | **Forward:** 5'- CACTACAGGCTCCGAGATGAACAAC -3' |
|  | **Reverse:** 5'- TGTCGTTGCTTGGTTCTCCTTGTAC -3' |
| IL-6 | **Forward:** 5’- CTCCCAACAGACCTGTCTATAC -3’ |
|  | **Reverse:** 5’- CCATTGCACAACTCTTTTCTCA -3’ |
| TNF-α | **Forward:** 5’- ATGTCTCAGCCTCTTCTCATTC -3’ |
|  | **Reverse:** 5’- GCTTGTCACTCGAATTTTGAGA -3’ |
| α-SMA | **Forward:** 5'- CGTGGCTATTCCTTCGTGACTACTG -3' |
|  | **Reverse:** 5'- CGTCAGGCAGTTCGTAGCTCTTC -3' |
| TGF-β1 | **Forward:** 5'- CCAGATCCTGTCCAAACTAAGG -3' |
|  | **Reverse:** 5'- CTCTTTAGCATAGTAGTCCGCT -3' |
| TGF-β2 | **Forward:** 5'- CTCGACATGGATCAGTTTATGC -3' |
|  | **Reverse:** 5'- ATAAACCTCCTTGGCGTAGTAC -3' |

**Rat abdominal aorta injury**

For closure of abdominal aorta defects with casein hydrogel in vivo, male Sprague Dawley rats weighing 320±20 g were adopted. In brief, rats were deep anesthetized with pentobarbital. After that, the abdominal aorta was separated and clipped in the two sides by artery clamps, and an incision was created. Next, casein hydrogel precursor solution was applied and illuminated for 3 min. Last, the distal and proximal clamps were successively removed, and the abdominal aorta was inspected for up to 4 min to detect bleeding.

**Porcine skin Injury**

A male Yorkshire swine weighing 30 kg was preanesthetized with telazol (4 mg/kg IM), and then anesthetized with inhaled isoflurane during the procedure. The animal was intubated and ventilation was maintained between 10 and 14 breaths/min with tidal volumes between 300 and 500 mL. Wounds were generated by a 6 mm biopsy to the posterior thigh. After removal of biopsied tissue, an incision of 1 cm deep was made with a 6 mm biopsy. The wound was allowed to bleed for approximately 10 s to ensure normal bleeding. Then, the hydrogel precursor solution was immediately added dropwise to the wound with light irradiation to crosslink in-situ. The control group received no treatment after injury. All experiments were recorded by camera for each group.

**Results**

**（c）**

**（b）**

**（a）**


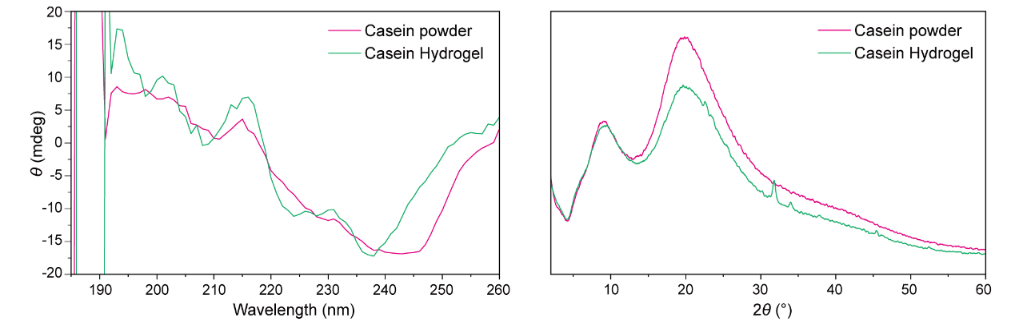

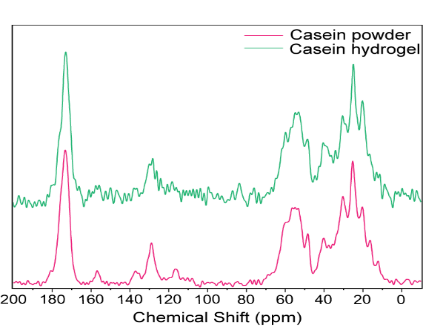


**Supplementary Fig. 1.** Structure of casein hydrogel. (a) Solid-state NMR spectra of casein powder and casein hydrogels. (b) Solid-state circular dichroism spectra of casein powder and casein hydrogels. (c) XRD profiles of casein powder and casein hydrogels.

**
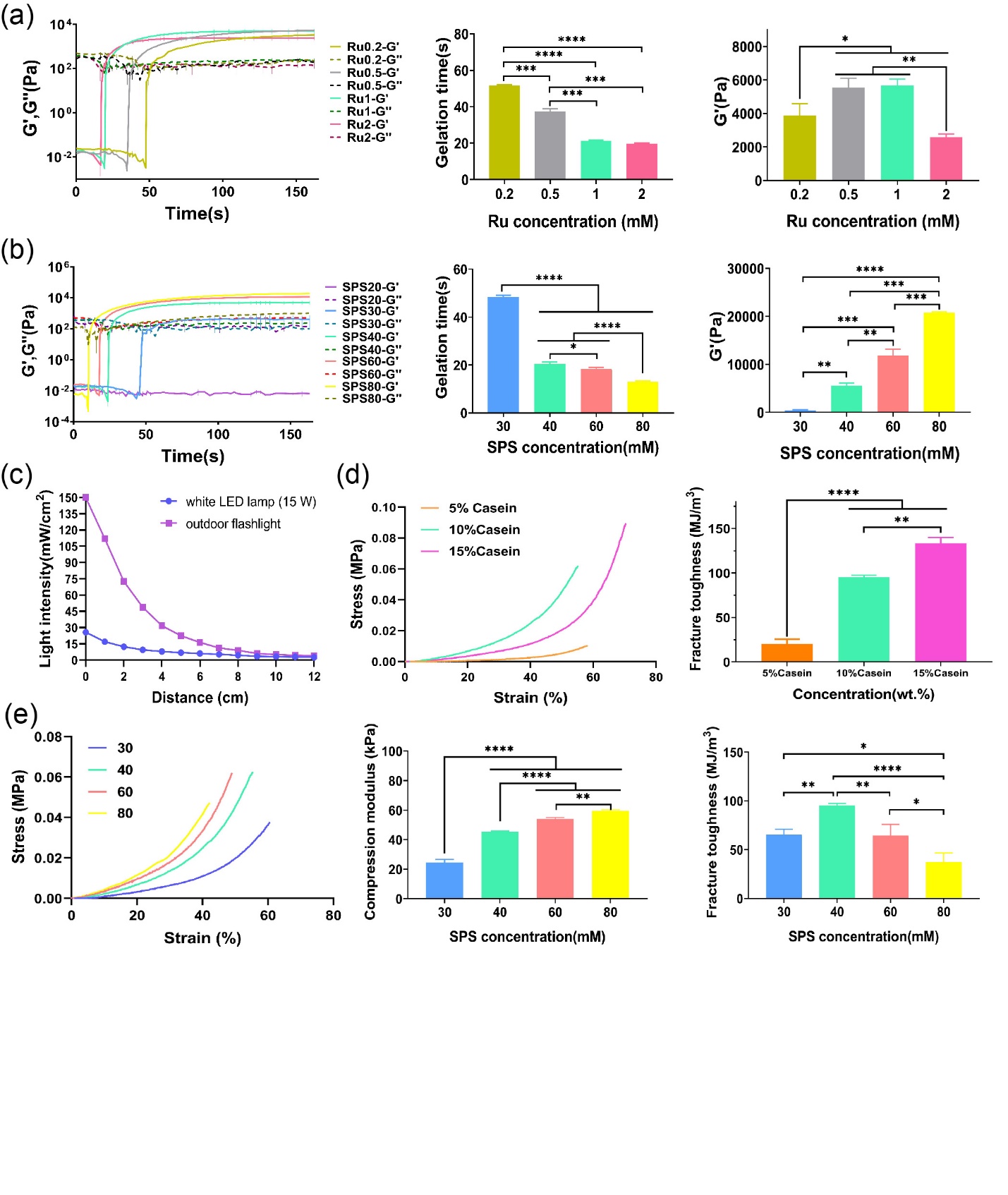
**

**Supplementary Fig. 2.** (a) Rheology analysis, gelation time and storage modulus of casein hydrogels with different Ru concentration (0.2, 0.5, 1.0, 2.0 mM), upon exposure to visible light for 160 s (n = 3); (b) Rheology analysis, gelation time and storage modulus of casein hydrogels with different SPS concentration (20, 30, 40, 60, 80 mM), upon exposure to visible light for 160 s (n = 3); (c) Light intensity at 450 nm with different distance to outdoor flashlight and white LED lamp (15 W); (d) Stress strain curves and fracture toughness of casein hydrogels with different casein concentration (n = 3); (e) Stress strain curves, compressive modulus and fracture toughness of hydrogels with different SPS concentration (30, 40, 60, 80 mM) (n = 3);

The effect of Ru concentration was first investigated by keeping the casein concentration constant at 10% (w/v) and SPS at 40 mM. The gelling point was at 51.90 ± 0.89 s (0.2 mM Ru) and 37.15 ± 0.42 s (0.5 mM Ru). These gelling points were lower than those for the formation of hydrogels containing 1 mM and 2 mM, which took 20.27 ± 0.68 s, and 19.63 ± 0.71 s, respectively (Supplementary Fig. 2a). The final storage modulus after complete gelation at 160 s was 5545.36 ± 421.65 Pa for 0.5 mM Ru and 5875.51 ± 223.18 Pa for 1 mM Ru, which was higher than the shear resistance of 3877.64 ± 325.52 Pa for 0.2 mM Ru and 2569.63 ± 217.11 Pa for 2 mM Ru. From these measurement results, 1 mM Ru was applied for subsequent experiments.

Then we investigated the effect of SPS concentration by keeping the casein concentration at 10% (w/v) and Ru at 1 mM. As shown in the Supplementary Fig. 2b, the higher the concentration of SPS (ranging from 30 ~ 80 mM) in the hydrogel precursor solution, the shorter the gel formation time and the higher the storage modulus at 160 s. When the SPS concentration was 20 mM, the rigid hydrogel was unformed, even if gelation occurred, the gel point was found to be very high, with a very small final storage modulus.

Supplementary Fig. 2c shows that the fracture toughness was 20.36 ± 6.85 MJ/m^3^ (5% casein), 95.17 ± 3.38 MJ/m^3^ (10% casein), and 133.47 ± 8.17 MJ/m^3^ (15% casein).

The compressive stress at break increased according to the SPS concentration, which was consistent with the storage modulus results in the rheological experiments (Supplementary Fig. 2d). Nevertheless, the SPS concentration has a different effect on the fracture toughness, with 40 mM SPS (95.17 ± 3.38 MJ/m^3^) showing significantly higher fracture toughness than 30 mM (65.33 ± 5.16 MJ/m^3^), 60 mM (64.63 ± 9.19 MJ/m^3^) and 80 mM (37.50 ± 9.68 MJ/m^3^) (Supplementary Fig. 2d). A particularly impressive outcome was observed for the 40 mM SPS.


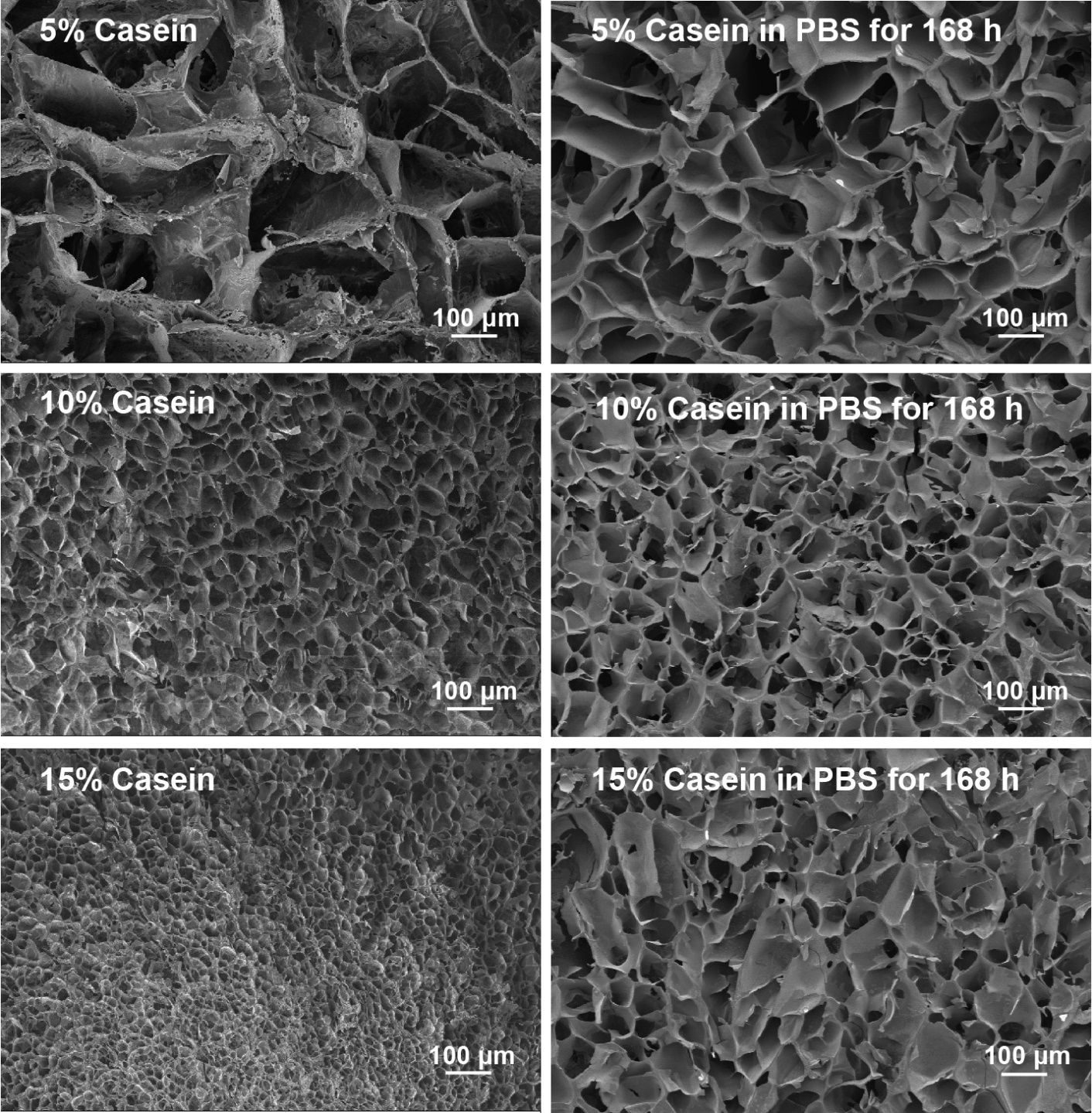


**Supplementary Fig. 3.** SEM images of hydrogels with different casein concentrations before and after swelling for 168 h, scale bar: 100 μm.


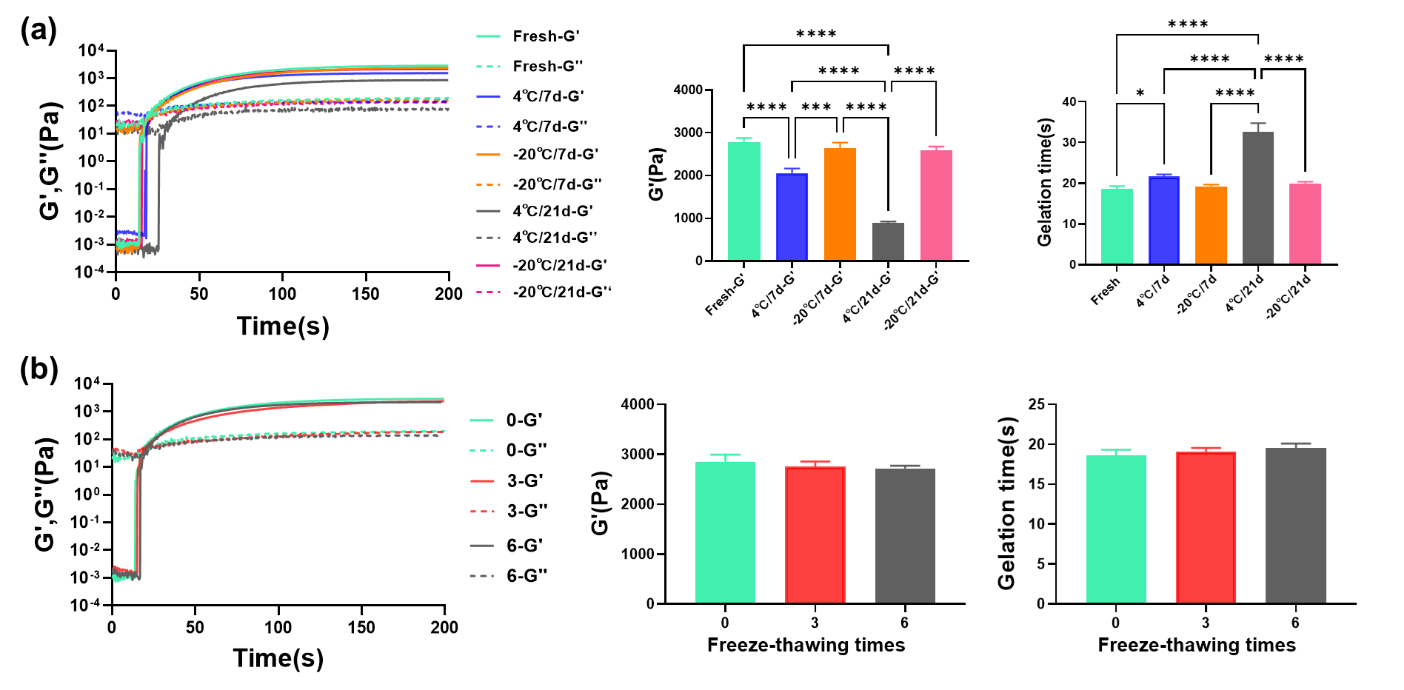


**Supplementary Fig. 4**. (a) Rheology analyses, gelation time and storage modulus of casein solution with different storage conditions, upon exposure to visible light at 450 nm (n = 3); (b) Rheology analyses, gelation time and storage modulus of casein solution with different freeze-thawing times, upon exposure to visible light at 450 nm (n = 3);

**
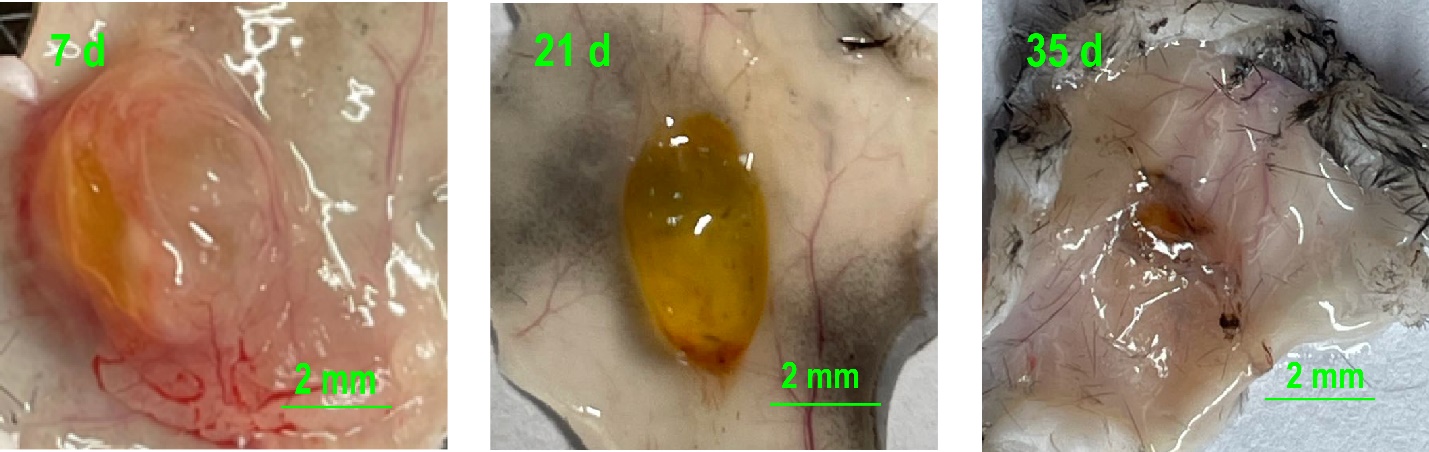
Supplementary Fig. 5.** Gross appearance of casein hydrogel after implantation.


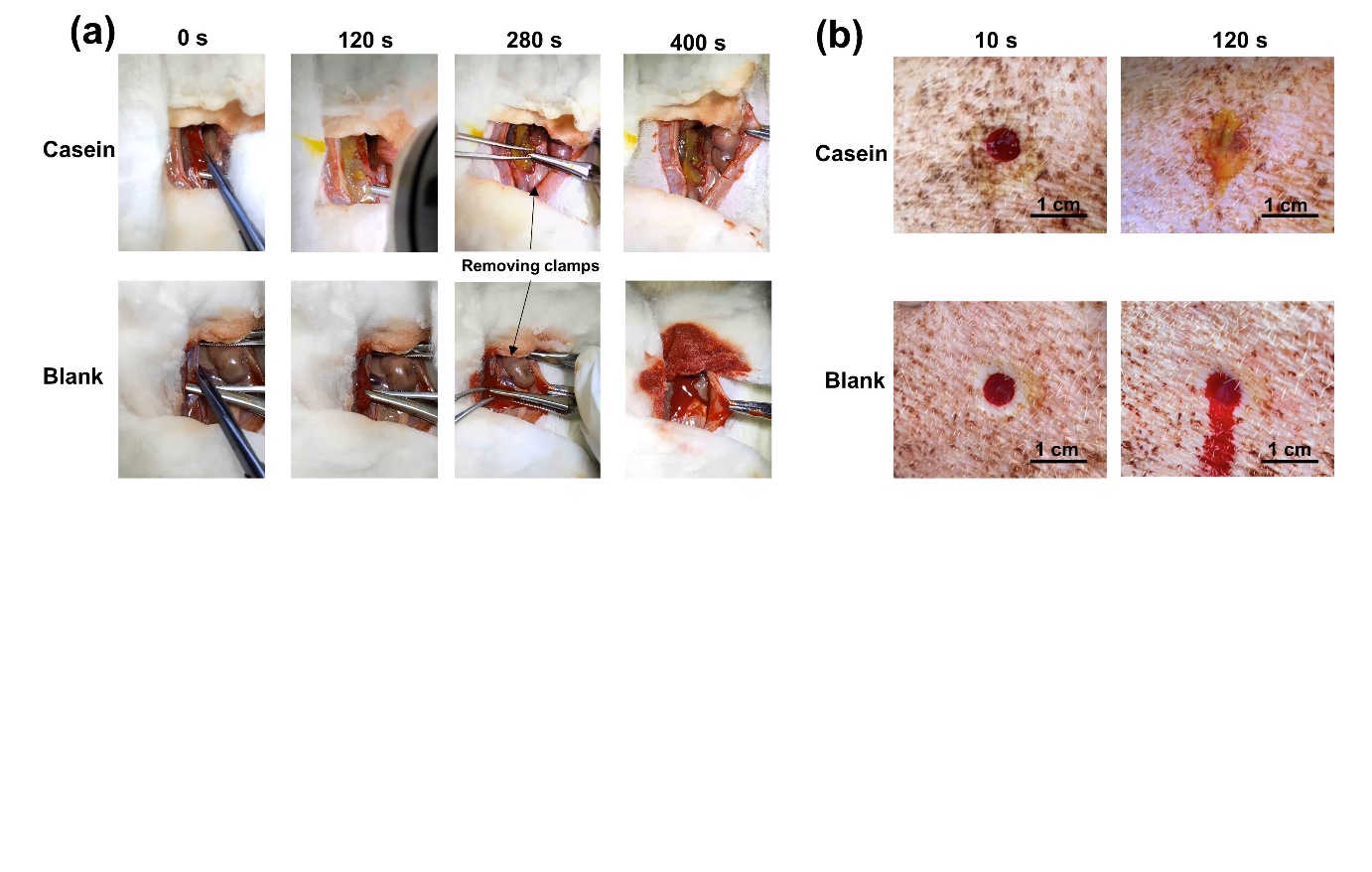


**Supplementary Fig. 6.** Application of casein hydrogel bioadhesive in a rat abdominal aorta injury model (a) and a porcine skin injury model (b).


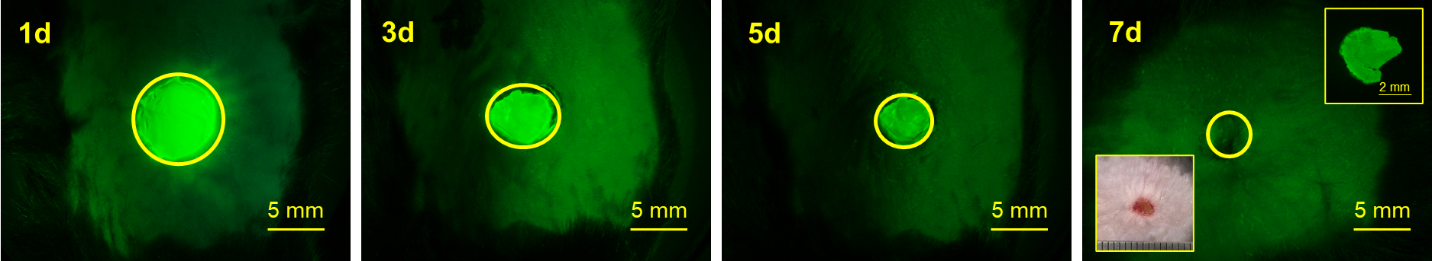


**Supplementary Fig. 7.** Fluorescence images of fluorescently-labeled casein hydrogel at the wound healing site at day 1, day 3, day 5 and day 7.


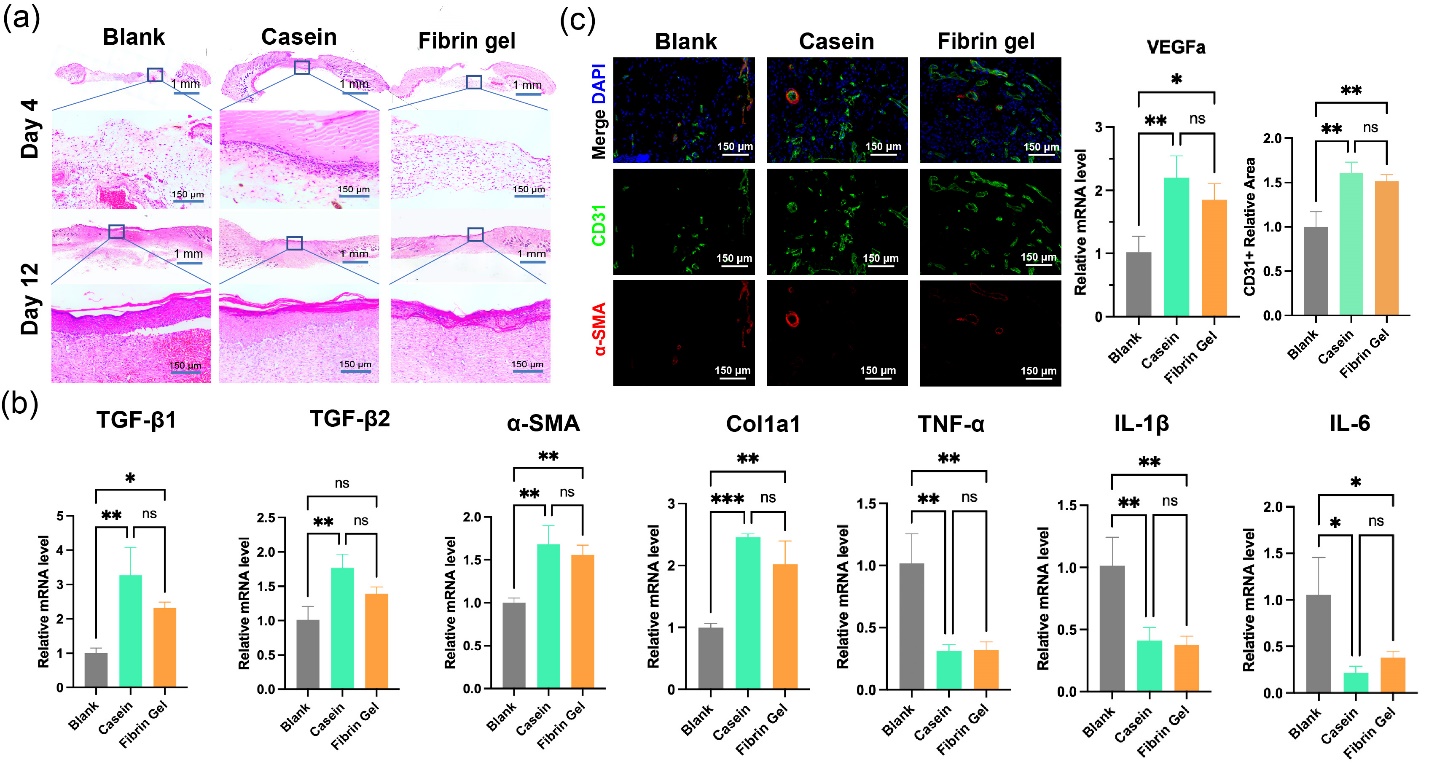


**Supplementary Fig. 8.** (a) Hematoxylin-eosin of wound tissues at day 4 and day 12; (b) Gene expression of TGF-β1, TGF-β2, α-SMA, Col1a1, TNF-α, IL-1β, IL-6 relative to the reference gene 18S in full-thickness wound tissues with different treatments at day 4 with qPCR (n = 3); (c) Immunofluorescence staining results of wound regeneration site with different treatment groups at day 4 with CD31 and α-SMA.

**Supplementary Movie 1**: The gelled casein hydrogel can adhere to dry paper and fail to adhere to wet tissue surface.

**Supplementary Movie 2**: The casein hydrogel bioadhesive effectively sealed a 5-mm-long strip wound in a porcine heart.

**Supplementary Movie 3**: The casein hydrogel bioadhesive bonds two pieces of pigskin soaking in PBS.

**Supplementary Movie 4**: Fibrin gel fails to bond two pieces of pigskin in cross-section.

**Supplementary Movie 5**: The casein hydrogel bioadhesive bonds two pieces of pigskin in cross-section.

**Supplementary Movie 6**: The hemostasis process of the casein hydrogel bioadhesive-treated groups in the mouse liver hemorrhage model.

**Supplementary Movie 7**: The hemorrhage process of the untreated groups in the mouse liver hemorrhage model.

**Supplementary Movie 8**: The hemostasis process of the fibrin gel-treated groups in the mouse liver hemorrhage model.

**Supplementary Movie 9**: The hemostasis process of the casein hydrogel bioadhesive -treated groups in the ear artery hemorrhage model of rabbits.

**Supplementary Movie 10**: The hemorrhage process of the untreated groups in the ear artery hemorrhage model of rabbits.

**Supplementary Movie 11**: The hemostasis process of the fibrin gel-treated groups in the ear artery hemorrhage model of rabbits.

**Supplementary Movie 12**: The hemostasis process of the untreated groups in the rat abdominal aorta injury model.

**Supplementary Movie 13**: The hemostasis process of the casein hydrogel bioadhesive -treated groups in the rat abdominal aorta injury model.

**Supplementary Movie 14**: The hemostasis process of the untreated groups in the porcine skin injury model.

**Supplementary Movie 15**: The hemostasis process of the casein hydrogel bioadhesive -treated groups in the porcine skin injury model.

**Supplementary Movie 16**: The casein hydrogel bioadhesive is gelled *in situ* with a cellphone flash.

**Supplementary Movie 17**: The casein hydrogel bioadhesive is gelled *in situ* with the light source of endoscope.
